# The role of inflammation in autoimmune disease: a therapeutic target

**DOI:** 10.3389/fimmu.2023.1267091

**Published:** 2023-10-04

**Authors:** Yu Xiang, Mingxue Zhang, Die Jiang, Qian Su, Jianyou Shi

**Affiliations:** ^1^ Department of Pharmacy, Personalized Drug Therapy Key Laboratory of Sichuan, Sichuan Academy of Medical Science & Sichuan Provincial People’s Hospital, Sichuan Provincial People’s Hospital, School of Medicine, University of Electronic Science and Technology of China, Chengdu, China; ^2^ State Key Laboratory of Southwestern Chinese Medicine Resources, Chengdu University of Traditional Chinese Medicine, Chengdu, Sichuan, China; ^3^ School of Life Science and Engineering, Southwest Jiaotong University, Chengdu, China; ^4^ Department of Health Management & Institute of Health Management, Sichuan Provincial People’s Hospital, University of Electronic Science and Technology of China, Chengdu, China

**Keywords:** autoimmunity, inflammation, pro-inflammatory factors, T cells, B cells

## Abstract

Autoimmune diseases (AIDs) are immune disorders whose incidence and prevalence are increasing year by year. AIDs are produced by the immune system’s misidentification of self-antigens, seemingly caused by excessive immune function, but in fact they are the result of reduced accuracy due to the decline in immune system function, which cannot clearly identify foreign invaders and self-antigens, thus issuing false attacks, and eventually leading to disease. The occurrence of AIDs is often accompanied by the emergence of inflammation, and inflammatory mediators (inflammatory factors, inflammasomes) play an important role in the pathogenesis of AIDs, which mediate the immune process by affecting innate cells (such as macrophages) and adaptive cells (such as T and B cells), and ultimately promote the occurrence of autoimmune responses, so targeting inflammatory mediators/pathways is one of emerging the treatment strategies of AIDs. This review will briefly describe the role of inflammation in the pathogenesis of different AIDs, and give a rough introduction to inhibitors targeting inflammatory factors, hoping to have reference significance for subsequent treatment options for AIDs.

## 1 Introduction

When talking about autoimmune diseases (AIDs), it is necessary to mention the concept of “immune tolerance”, which is an acquired feature during human development (1). Immune tolerance is disrupted by immune system disorders leading to malignant proliferation of autoreactive T and B lymphocyte populations, which in turn produce an attack response to autoantigens (2, 3). This process is the basis and root cause of AIDs, mainly caused by the immune system’s ineffective judgment of self and non-self (4). The occurrence of AIDs usually goes through three stages, first of all, the immune system is blocked under the stimulation of a variety of factors, at which time the immune tolerance has been destroyed. In this process, the activated innate immune response triggers the emergence of adaptive immune response, and T and B cells misrecognize antigens to cause abnormal immune function; Secondly, abnormal proliferation of innate immune cells (macrophages, granulocytes, dendritic cells) secretes a large number of inflammatory factors to stimulate abnormal infiltration of T and B cells, and eventually patients has progressive inflammation and tissue damage; Finally, the control stage of AIDs is usually limiting the development of autoimmune responses from the internal and extrinsic mechanisms of cells, and this stage would continue to have the possibility of remission and recurrence (5, 6).

In terms of maintaining immune homeostia and preventing immune tolerance, regulatory T cells (Treg cells) are a key cell that reduces the activation and proliferation of autoreactive T cells in the body through cell-to-cell contact and secretion of inhibitory cytokines in various immune cell subsets mediated by the regulatory factor Foxp3, one of the key transcription factors for Treg cell development and function, thereby alleviating the development of AIDs (7). However, due to the changes of Foxp3 or epigenetics, Treg cells might be unstable or plasticity (TH1-like, TH2-like or TH17-like cells) to develop numerical or functional deficits, leading to AIDs (8). Therefore, maintaining the balance between autoimmune effects and immunomodulatory responses is pivotal to treating AIDs (6, 9). Next, this review mainly discusses the role of inflammation in AIDs and proposes therapeutic strategies targeting inflammatory mediators/pathways.

## 2 Autoimmune diseases

### 2.1 Features and classification

Autoimmune reactions are physiological and pathological, and physiological autoimmunity is usually a low-level recognition of exogenous antigens by immune cells - T cells and B cells. Because autoantigens have similarities with foreign antigens, the specific recognition of the two is not well distinguished, so it leads to the emergence of pathological autoimmunity, which is accompanied by a decrease in the survival rate and activation threshold of B cells, as well as changes in T cell activation and proliferation, which also marks immune tolerance disorders (5, 10). The main feature of AIDs is the presence of autoantibodies targeting the bulk tissue, which would trigger its own cytotoxic reaction, eventually resulting in pathological changes in organ tissues, accompanied by inflammation, which is also called pathological AIDs (2, 4). Helper T cells produce cytokines or recruit inflammatory cells to cause tissue damage, while autoantibodies cause cells’ damage or death and drive inflammation through mechanisms of interaction with their antigen-binding sites (Fab) or crystallizable fragments (Fc), formation of immune complexes, cytolysis or phagocytosis of target cells, both of which mediate the emergence of AIDs (5, 10).

At present, there are more than 100 AIDs, including rheumatoid arthritis, Sjogren’s syndrome, systemic sclerosis, juvenile idiopathic arthritis, psoriasis. An important way to classify AIDs is through systemic and organ-specific distinctions, of which systemic representatives are systemic lupus erythematosus, which occurs in joints, kidneys, lungs, skin, and heart (2). Such diseases may have similarities in clinical, immunological and genetic characteristics, while organ-specific representatives are type 1 diabetes mellitus that occurs in the pancreas, but different AIDs have specific disease characteristics (2, 11).

### 2.2 Epidemiology and diagnosis

For the general population, the prevalence of AIDs is about 4.5%, of which 2.7% in men and 6.4% in women with significant differences. The risk of most AIDs in women is much higher than in men, indicating a bias of AIDs towards women (12). However, there is a higher proportion of some disorders in men, including Guillain-Barré syndrome and ankylosing spondylitis, which show a higher prevalence than in women (13). The main reason for the gender differences in the manifestations of AIDs may be discrepant in the immune systems of men and women, in which diverse categories of lymphocytes are different. Women have more T lymphocytes and show a stronger autoimmune response, which might make women more susceptible to AIDs (14, 15). In addition, the prevalence of Ulcerative colitis (UC) and Crohn’s disease (CD) is at a balanced level in the proportion of men and women, so there are geographical differences in the incidence and prevalence of different AIDs in men and women. For example, celiac disease usually occurs more in women, but shows a higher prevalence in men in India (16).

Currently, clinical symptoms, physical examination, laboratory tests, and radiological results are fundamental to the diagnosis of AIDs. In the case of rheumatoid arthritis, the physical examination focuses on joint pain, swelling, redness, and rigidity, and laboratory tests include inflammatory and serological markers (5, 17). In most AIDs, an antinuclear antibody (ANA) test can initially screen suspected patients. A positive result indicates that the immune system is under false immune stress, and the higher the number, the greater the probability of developing the autoimmune disease, but there are false positives (18). Hence, once positive is confirmed, antibody tests are also performed, combined with clinical features to obtain more accurate diagnostic information (19).

The reactivity of binding autoantibodies in serum of autoimmune patients is a key step in diagnosis, and autoantibodies have been initiated as to be used as a biomarker in the diagnosis of some diseases. For instance, autoantibodies against SSA and SSB in Sjogren’s syndrome (3), anti-PLA2R antibodies in primary membranous nephropathy (20), and IgM anti-dsDNA antibodies to prevent lupus nephritis (21). These predictive antibodies might be able to recognize the presence of risk of AIDs and play a preventive role in risk factors (22). Meanwhile, citrullineated products, including CPs and citrullineated proteins, began to be used as markers for the diagnosis of RA. CPs were detected by synovial samples from inflammatory joints in RA patients, while anti-citrullinated peptides/protein antibodies (ACPAs), which could be converted to citrulline by PADs enzymes, disrupting immune tolerance, could be detected by mass spectrometry (17). In addition, RA-related autoantibodies rheumatoid factor (RF) are also an indicator of laboratory testing, but diagnosis must be made in conjunction with imaging (23). Therefore, the autoantibody immune reactivity in the patient is important diagnostic information, which has reference significance for some potential immune diseases. Autoantibody detection experiments should be carried out on the basis of some other test results and clinical features, and finally combined with a variety of test results to obtain diagnostic conclusions (3, 19).

In addition, inflammatory factors may play a role in the diagnosis and treatment of diseases by acting as biomarkers of inflammatory diseases to assess the degree of activity. For example, integrin is a key pathogenesis in the mechanism of juvenile idiopathic arthritis, and elevated level of it is an important marker for patients. Meanwhile, testing for CXCL9 may be a useful test for this disease activity (24). Measurement for serum levels of cytokines or soluble cytokine receptors may make a judgment about the efficacy of biologics in patients. Nishina et al. found that baseline levels of IL-6R appear to predict clinical remission after tocilizumab treatment in RA patients, but are not associated with disease activity (25). Therefore, inflammatory factors are not only important players in the pathogenesis of AIDs, but also have an auxiliary role in diagnosis and treatment.

### 2.3 Causative factors

The genetic susceptibility to AIDs may be related to the incidence and risk of diseases. Studies have shown the prevalence of first-degree family members and monozygotic twins of patients, and the matching rate of monozygotic twins is higher than that of monozygotic twins. The reason is probably the genes of such people are too similar to the genes of infected people, and then the probability of carrying disease genes would be higher. so the risk of disease would increase, indicating vulnerability to these diseases must be rooted at least in part in heredity (10). AIDs, on the other hand, are often the result of multiple susceptibility genes leading to an abnormal phenotype. At the same time, the presence of susceptibility genes makes gene polymorphisms promote autoimmunity (10). According to genomic analysis, gene mutations and polymorphisms are strongly associated with the development of AIDs. For example, the correlation between HLA-DR3, a class II HLA molecule, and autoantibodies, might affect subtypes of systemic lupus erythematosus, Sjogren’s syndrome, and autoimmune myositis (11). The emergence of susceptibility is frequently connected with risk factors including smoking, obesity, family history of AIDs, immune deficiency, and low vitamin D status, so these aspects could be used to avoid it when considering preventive measures for the disease to reduce the probability of the disease (22).

Nevertheless, the occurrence of AIDs is caused by a combination of genetic susceptibility and environmental factors of to contribute an imbalanced response of the immune system between self-defense and immune tolerance (2, 4). Environmental factors also play a crucial role, for example, cutaneous lupus might be caused by excessive apoptosis due to ultraviolet radiation, which possibly results in the production of autoantigens to trigger an autoimmune response (26). Meanwhile, genetic and environmental factors interact with each other, for example, smoking may contribute to the production of autoantibodies in autoimmune myositis, which is the result of interaction with HLA haplotypes (11). Therefore, immune-related gene polymorphisms may lower the threshold for autoreactive T cell activation, which combined with environmental stimulation and improper regulation of cytokines to lead to tissue damage ultimately.

## 3 Autoimmune diseases and autoinflammatory diseases

The most essential difference between autoimmunity and autoinflammation is that the type of immune system disorder is not the same (27). First of all, it is necessary to understand two concepts, innate immunity and adaptive immunity, the former is the first barrier against injury and infection, mainly involving monocytes, macrophages, neutrophils, but less specificity, while the latter has a stronger resistance but takes more time to appear, produced by innate immune stimulation (28). Both activate the conduction of TNF, IL and IFN signaling pathways, but overactivation carries a risk of autoimmunity and autoinflammation (29). Autoimmunity is an adaptive autoimmunity, the major body involved is lymphoid T and B cells, mainly after the autoimmune tolerance is disrupted and the immune system dysfunction appears a sustained immune response to its own cells, which in turn leads to tissue damage and clinical features (30).

However, AIDs and autoinflammatory diseases are similar and potentially linked. Both diseases can cause systemic injury, although the pathways leading to tissue damage are different, autoinflammatory diseases are inflammation and damage directly caused by the innate immune system, while AIDs lead to the persistence of inflammation through the corresponding pathway after the emergence of adaptive immunity caused by innate immunity (28). However, the emergence of adaptive immunity involves innate immunity, and long-term stimulation of congenital inflammation contributes to abnormal activation and infiltration of T and B cells, which disrupts immune tolerance and leads to the production of autoantibodies, resulting in autoimmunity to aggravate tissue damage and inflammation (28). In the meantime, both innate immunity and adaptive immunity are affected by the cytokine IL-1β. The former manifests IL-1β, as a driver of inflammation, might lead to innate immune abnormalities to result in the emergence of autoinflammation (31), while the latter is an increase in proliferation of lymphoid T and B cells due to the impact of IL-1β, which possibly increases adaptive immunity, and if this process is excessive, it might lead to the development of AIDs (32). Hence, the emergence of AIDs might be accompanied by the appearance of features of autoinflammation, and the demarcation between the two is not very well defined clinically. So there are three situations in the pathogenesis of immune diseases, namely simple autoimmune mechanism, complete autoinflammatory mechanism and autoinflammatory-autoimmune mechanism, and clarifying the specific pathogenesis is very critical for the treatment of the disease (27).

At present, the treatment strategies of AIDs focus on targeting lymphocytes, and anti-inflammatory strategies have good results in the treatment of autoinflammatory diseases. From the perspective of pathogenesis, these related cytokines and inflammatory complexes also play an important role in AIDs. For example, a significant increase in IL-18 levels was found in the serum of patients with systemic lupus erythematosus, and its expression also correlated with the intensity of damage and renal activity in patients (33). The innate immune system of rheumatoid arthritis patients was activated, so the macrophages involved in it released the pro-inflammatory factors TNF, IL-1β, IL-8, and the inflammatory process indicated that the nlrp3 inflammasome was abnormally activated, which possibly drove the stimulation of adaptive immunity, potentially leading to autoimmune production (34, 35). These suggest that inflammatory processes play an important role in autoimmune responses, and that anti-inflammatory strategies might become another effective therapeutic measure for AIDs.

## 4 The role of inflammation in the pathogenesis of autoimmune diseases

When the body is subjected to external adverse stimuli, it will stimulate the body’s innate immunity and trigger inflammation, followed by the emergence of adaptive immunity. Once the adaptive immune system is disordered, it may lead to AIDs. The microenvironment balance of pro-inflammatory and anti-inflammatory cytokines in these processes is closely associated with AIDs, particularly rheumatoid arthritis, inflammatory bowel disease, and systemic lupus erythematosus, which have a persistent inflammatory response in the pathological features of AIDs (36, 37). Therefore, inflammatory dysfunction plays an important role in the pathogenesis of AIDs. Subsequently, inflammation may become the treatment direction of the disease. However, there are many AIDs, and the role of inflammation in different diseases may be different. The potential role of inflammation in the pathogenesis of different AIDs will be briefly introduced below.

### 4.1 Rheumatoid arthritis

**Figure 1:**
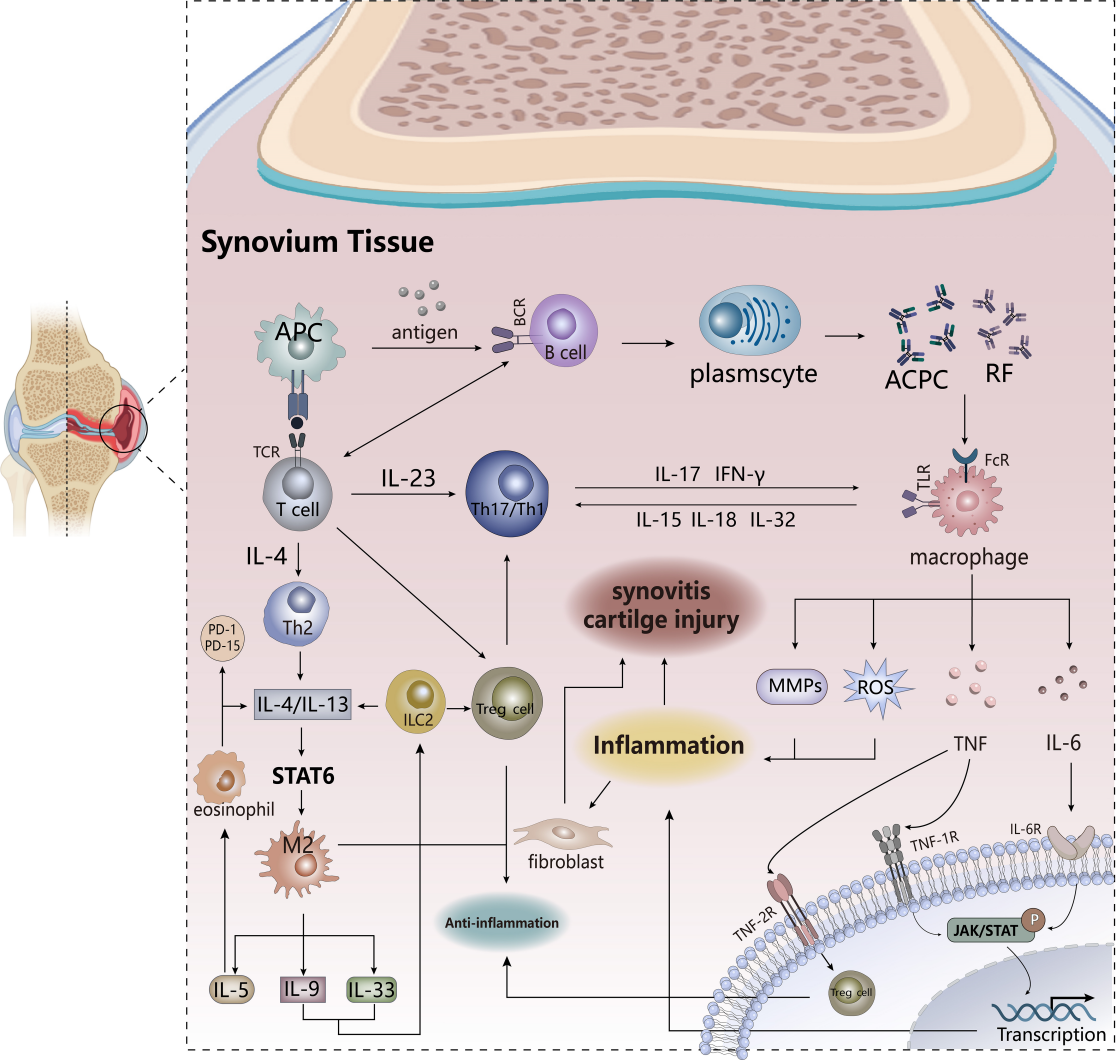
The pathogenesis of RA. Antigen-presenting cells activate T cells and B cells to trigger adaptive immunity. B cells produce autoantibodies that stimulate macrophages to secrete pro-inflammatory factors and promote transcription of inflammatory genes. T cell differentiation into TH17 cells plays a pro-inflammatory role, and IL-4/IL-13 produced by TH2 cells triggers the activation of anti-inflammatory signaling pathways, and the production of anti-inflammatory factors and anti-inflammatory lipids is conducive to disease reversal. PD1 PD15, anti-inflammatory lipids. APCs, antigen-presenting cells. TCR, T cell receptor. TLR, Toll-like receptor.

Rheumatoid arthritis (RA) (**Figure 1**) is a chronic inflammatory autoimmune disease characterized by synovitis that clinically presents with joint swelling and pain, cartilage erosion, and injury, accompanied by a persistent inflammatory state (38). RA is usually caused by immune cells soaking the membrane joints. The occurrence of synovitis is induced by the infiltration of a large number of white blood cells into the synovial compartment, which is related to immune activation. Under the combined action of innate and adaptive immune systems, heterogeneous changes in stromal cells (fibroblasts) in the synovium result in RA (39, 40). Macrophages polarize to the M1 pro-inflammatory phenotype and produce a large number of pro-inflammatory cytokines (such as TNF, IL-1 and IL-6) and pro-inflammatory molecules or mediators (such as inflammasomes, reactive oxygen species, MMPs) to promote the ongoing inflammatory process and activate neighboring T cells, dendritic cells, fibroblast-like synovial cells (FLS), ultimately leading to joint cartilage damage (39, 41); the M2 anti-inflammatory phenotype is far from sufficient to resist the deterioration of inflammation (41). In the remission of RA, a cluster of macrophages, MerTK^pos^CD206^pos^, has a recovery effect on inflammation and induces the repair capacity of FLS, which probably helps maintain immune homeostasis in the joints (40), so the bidirectional action of macrophages works at different stages. Adaptive immune cells (such as T-helper-1 and T-helper-17 cells, B cells) begin diffuse infiltration into the synovium, and gradually proliferate, differentiate and produce autoantibodies, which also produce inflammation-related effector factors (such as IL-10, IL-17) and recruit inflammatory cells (37). This process is accompanied by selective activation of aggressive synovial fibroblasts, which produce pro-inflammatory factors and induce the transition from joint inflammation to chronic synovitis, while accelerating the migration of synovitis to other joints, driving synovial inflammation and bone erosion (39, 42–44). Hence, the inflammatory state has always been accompanied by the development of RA, and the degree changes with different stages, from the initial arthritis to chronic synovitis, and may continue to worsen.

ACPAs produced by B cells and rheumatoid factor (RF) are the hallmark autoantibodies in RA patients, the former is significantly more specific in patients than the latter, and plays a key role in the autoimmune response (42, 45). Individuals with high expression of ACPAs and RF develop acute arthritis for a short time but resolve quickly, and yet, there is still a possibility of developing chronic synovitis. Under the induction of ACPAs, osteoclasts secrete CXCL8 to promote neutrophil differentiation and infiltration into the synovial compartment. ACPAs and the immune complex between ACPA-IgG stimulates macrophages to produce pro-inflammatory factors to drive inflammation by binding to Toll-like receptor 4 (TLR4) and Fc receptors (42). In addition, complement activation or microvascular damage may alter vascular permeability to accelerate the transfer of inflammatory cells to the synovium, promoting the progression of RA (45). Thus, ACPAs and RF-mediated events promote the activation of inflammation-associated cytokines, increasing the damaging effects of inflammation and driving the development of chronic synovitis (39). There are also studies that show in leukocyte-rich RA, levels of inflammatory response genes (PTGS2, PTGER3, and ICAM1) in fibroblasts and monocytes are significantly elevated (46). The above shows that inflammation is an important player in RA, and what are the effects of inflammatory factors and inflammatory mediators produced by these cells on RA?

Up to now, there have been two pro-inflammatory factors in the pathogenesis of RA, TNF and IL-6, which are the most studied. Both of them play a multifaceted role in the pathogenesis of RA, which stimulate the activation of stromal cells to aggravate the inflammatory response. TNF activates NF-kB and induces transcription of downstream inflammatory target genes through binding to TNF1R, and also promotes the recruitment of immune cells to the site of inflammation to accelerate tissue damage, while binding to TNF2R mediates the function and differentiation of Treg cells to maintain immune homeostasis (47). IL-6, mainly derived from Subliming fibroblasts and B cell (46), activates the intracellular JAK/STAT signaling pathway by binding to receptors, and STAT phosphorylated by JAK translocates to the nucleus to mediate the transcription of target genes, affecting cell proliferation and differentiation (47, 48). This signaling pathway exhibits constitutive phosphorylation activity in both T cells and monocytes. If this signaling pathway is impaired, it could effectively alleviate and improve the progression of RA (49), so JAK inhibitors have good therapeutic prospects in RA patients. IL-6 also stimulates CD4+ T cell proliferation and differentiation of Treg, Th17, and Tfh cells (47). Granulocyte-macrophage colony-stimulating factor (GM-CSF), a hematopoietic growth factor produced primarily by T cells and stromal cells, acts as a soluble pro-inflammatory factor that can lead to inflammation by stimulating innate immune cells, such as inducing the polarization of the macrophage M1 phenotype and stimulating the activation of neutrophils (47, 50). Some research also indicates that MMPs, highly expressed in RA patients, are derived from a variety of cells, particularly cadherin-11-positive FLS, where proteases such as collagenase and matrix lysin cause severe damage to cartilage (39). These pro-inflammatory factors activate FLS to release more cytokines, resulting in the recruitment of a large number of pro-inflammatory factors in the synovial space, which extremely increases the number of such cytokines and stimulates the formation of osteoclasts and the degradation of cartilage. The synergistic effect between pro-inflammatory factors is required for the pathogenesis of RA, such as the stimulating effect of TNF-α on IL-6 and the IL-6-STAT pathway on IL-17-induced inflammation (47).

At the same time, some anti-inflammatory factors play a role in disease alleviation in RA, including IL-4, IL-13, IL-5, IL-9, and IL-33 (47). IL-4 and IL-13, mainly produced by helper T cells 2 (TH2) and 2 groups of innate lymphoid cells (ILC2s), activate the downstream STAT6 pathway by binding to the receptor to promote the polarization process of the macrophage M2 phenotype, accelerating the release of other anti-inflammatory factors, and inhibit the infiltration of inflammatory cells into the synovium and the production of pro-inflammatory factors. These effects reduce the production of osteoclasts and the damage of chondrocytes, so ultimately the tissue damage and inflammation of RA are alleviated (47). The anti-inflammatory effect of IL-5 is mainly manifested in the recruitment of eosinophils at the site of inflammation, and the cells help the resolution of inflammation by the production of IL-4 and IL-13 to mediate the differentiation of the M2 phenotype and the secretion of anti-inflammatory lipids (such as PD1, PD15) (47, 51). The anti-inflammatory action of IL-9 occurs mainly during the regression phase of RA, which affects the proliferation of ILC2 to make Treg cells be activated, and this regressive role on arthritis reduces cartilage damage to relieve inflammation and maintain immune homeostasis (52). The function of IL-33 on RA varies with the stage of the disease. In the early stage, it plays a pro-inflammatory role by promoting the migration of inflammatory cells and the release of related factors, while in the regression phase of RA, IL-33 affects the proliferation and differentiation of ILC2 and TH2 cells, as well as the tendency to regulatory M2 phenotypic production, especially the activation of Treg cell population, which are very beneficial for reversing RA (53).

In summary, most anti-inflammatory cytokines indirectly or directly mediate the polarization process from macrophage M1 to M2 phenotype, which ultimately influences tissue damage and inflammation. However, there are more than two phenotypes of macrophages, and the distribution of polarized macrophage subsets varies in different diseases. Studies have shown higher expression of CD163 in synovitis in spondylarthritis compared with RA, which might lead to different outcomes in chronic synovitis (54). In addition to being an autoimmune disease, RA is also a chronic systemic inflammatory disease, in which inflammation is the main pathological feature. Consequently, figuring out the role of inflammation in RA is very beneficial to the development of anti-cytokine therapeutic agents. Anti-inflammatory therapy may become the first choice for this disease in the future, which has two research ideas, namely inhibitors of pro-inflammatory factors or agonists of anti-inflammatory factors, but which treatment of RA is better needs further research.

### 4.2 Systemic lupus erythematosus

**Figure 2:**
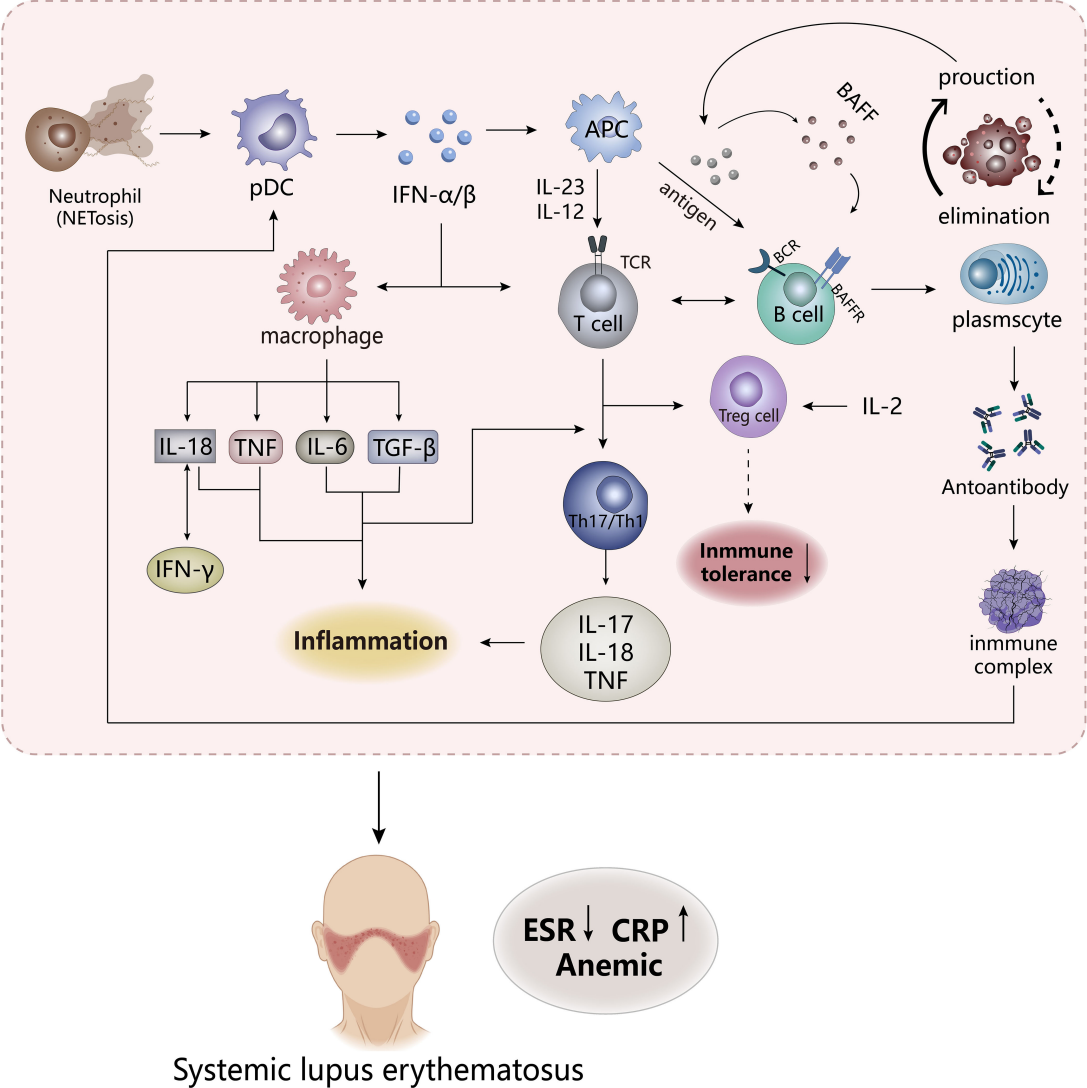
The pathogenesis of SLE. The appearance of NETosis in neutrophils promotes the secretion of IFN-α/β by pDC cells to stimulate the body’s innate immunity and adaptive immunity, while the frontal interaction between inflammatory cells and lymphoid T and B cells results in the production of a large number of inflammatory factors. APCs, antigen-presenting cells. TCR, T cell receptor. BCR, B cell receptor. BAFFR, B-cell activating factor receptor. CRP, C-reactive protein; ESR, erythrocyte sedimentation rate.

systemic lupus erythematosus (SLE) (**Figure 2**), as a systemic autoimmune disease, is also a chronic diffuse connective tissue disease that invades the systemic system, which often occurs in women, and clinically manifests skin lesions, arthritis, kidney disease, hematologic changes, with a great risk of cardiovascular morbidity (55). The main feature of SLE is that the process of destruction of immune tolerance is accompanied by the emergence of autoantibodies and immune complexes, which lead to the dysfunction of T cells and B cells and the abnormal increasement in some cytokines (56). Such disease usually leaves most organs in an inflammatory state and tissue damage, and the degree of inflammation of each organ is often used as an important reference indicator for SLE activity scores, including the level of C-reactive protein (CRP), a standard marker of inflammation, decreased erythrocyte sedimentation rate (ESR) possibly triggered by inflammation and anemia (57, 58).

Studies have shown that most patients with SLE have “type I interferon (IFN) characteristics”, and its content and induced gene expression are elevated *in vivo*, which is highly related to the pathogenesis of SLE. So IFN is an important pathogenic factor leading to the destruction of immune tolerance in SLE. Previous studies have shown that IFN has antiviral function, which is a key linker of innate immune response and adaptive immune response. Under abnormal external stimulation, a large number of IFN-α/β produced would affect the activation and proliferation of immune cells (macrophages, CD8-T cells, B cells) and induce apoptosis of infected cells (59, 60). But in SLE, the imbalance in the production and clearance of apoptotic cells leads to an increase in autoantigens, which might be presented to autoreactive B cells to influence the body’s immune tolerance. These induce the emergence of pro-inflammatory factors and autoantibodies and an increase in immune complexes to lead to massive deposition of their various organs and tissues, which might stimulate the response of the autoimmune response to result in tissue damage and inflammation (61, 62). IFN-α, mainly derived from plasmacyte-like dendritic cells (pDC), could affect B cells in many ways, including stimulating dendritic cells to produce B-cell activating factor (BAFF, also known as BLyS), increasing the response of B cells to BAFF and promoting the transformation of B cells (63), which may increase the production of autoantibodies. Stimulation of Treg cell dysfunction by IFN-α may induce disruption of immune tolerance, but the regulation of Treg cells by IL-2 may reverse this phenomenon. In addition, IFN stimulates multiple cells to produce pro-inflammatory and chemokines. It could be seen that IFN plays an important role in the pathogenesis of SLE. Compared to macrophages in RA, neutrophils are an important factor driving early SLE inflammation and organ damage, which release proteases, ROS, and pro-inflammatory factors to stimulate immune disorders (64). Abnormal subsets have highly expressed NETosis, a cell death mechanism, which presents neutrophil extracellular traps (NETs), in which contain pro-inflammatory factors that promote the development of inflammation. At the same time, NETs as autoantigens also stimulate the emergence of anti-neutrophil cytosolic antibodies to form immune complexes, which promotes more IFN production (62, 65). The immune complexes formed by these processes are absorbed by phagocytes, DCs, and pDCs through the Fc receptor to activate autoreactive T and B cells in the immune system (6, 57). Immune complexes may be deposited in various organs if they are not effectively cleared, leading to tissue damage and inflammation (6).

The expression of many cytokines in SLE is at an increased level, which affects the destruction of susceptibility and tolerance of SLE (65). These factors are dysfunctional before the appearance of clinical features of SLE, of which IL-18 and TNF are the two most important pro-inflammatory factors. Both could be used as inflammatory markers of SLE, are extremely elevated in patients, and their expression has a great correlation with the degree of SLE activity (56). IL-18 and IFN-γ are positively correlated, possibly because IL-18 induces the production of IFN-γ, while IFN-γ in turn affects the expression of IL-18-binding proteins, so the synergy between these two factors promotes the development of SLE (56, 66, 67). However, IL-18-binding proteins may produce a negative feedback regulation to reduce the production of IL-18 and IFN-γ, which has been confirmed in some preclinical studies, and it may be a new idea in the treatment of SLE (67, 68). These proteins appear to play the role of inhibitors in IL-18 and IFN-γ. The role of TNF in SLE is currently controversial, although TNF is involved in autoimmune responses in a variety of pathways, including immunomodulatory effects through the effects on proliferation, differentiation and cytokine secretion of B-cells, T cells, and dendritic cells, and the pro-inflammatory effects on the aggregation of neutrophils and activation of monocytes, and the stimulation of IFN expression (69). Preclinical studies have shown that after administration of high doses of TNF-α, lupus-susceptible mice delayed disease onset without preventing the onset of disease (70), while TNF-α in mice already suffering from lupus might have malignant consequences (71), suggesting that the effect of TNF-α on lupus may vary depending on the state and tissue of the disease (i.e., the former is the lupus susceptibility model and the latter is the experimental SLE). These all show that the ambiguity of the effect of TNF-α on SLE, and how its interaction with the receptor would have on SLE is not very clear. Subsequent experiments are needed to explore, but the pro-inflammatory effect of TNF-α on inflammation in SLE is very clear (72). In addition, IL-17, as a pro-inflammatory factor, recruits inflammatory factors, chemokines, and inflammatory cells to the tissue site to affect inflammation and damage (65). The IL-23/IL-17 axis formed by its combination with IL-23 may be positively correlated with the severity of SLE, mainly because Th17 cells acting on IL-23 could produce IL-17 and expand it to drive the development of inflammation (73). Meanwhile, studies found that the IL-12/IL-23 axis appeared to play a role in SLE, and targeting this mediator may inhibit the progression of the disease. It could be seen that the inflammation of SLE is produced by the combined action of many factors.

### 4.3 Systemic sclerosis

**Figure 3:**
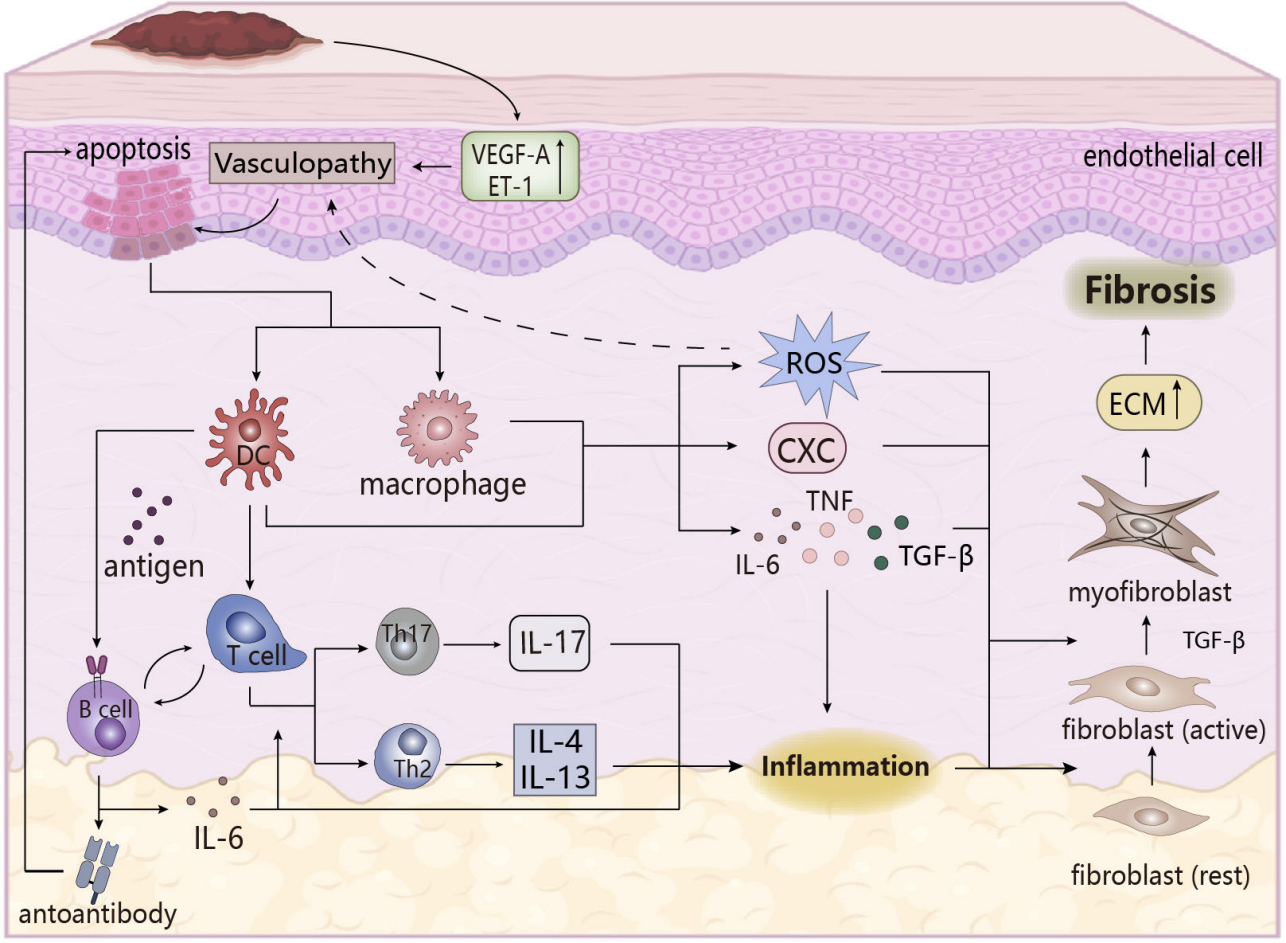
The pathogenesis of SSc. Upregulation of vasoactive factors affects apoptosis of epithelial cells, followed by cytokines that stimulate the immune response in the body, and the inflammatory response that occurs eventually triggers the transformation of fibroblasts, leading to the formation of skin fibrosis. ECM, extracellular matrix. CXC, chemotaxis.

Systemic sclerosis (SSc) (**Figure 3**), also known as scleroderma, is an autoimmune chronic fibrotic disease, clinically manifested as skin hardening of the limbs and face, essentially caused by severe skin fibrosis. In the early stage of the disease there is no obvious specific clinical feature, making early diagnosis very difficult, so it can only be judged by the Raynaud’s phenomenon due to endothelial dysfunction, autoantibodies and skin phenotype. In the terminal stage, the disease deterioration is more serious to be easily diagnosed, including ulceration of the fingers, joint contractures, sclerosis (74, 75). As a systemic disease, SSc is usually manifested in the kidneys, heart, gastrointestinal tract, and musculoskeletal disease, especially the emergence of interstitial lung diseases, which is the main cause of SSc’s death. Therefore, screening patients with SSc for organ involvement is critical (75).

The main pathological features in the pathogenesis of SSc are vascular lesions, immune system disorders, and skin fibrosis, which are closely related and accompanied by the emergence of early inflammation, an important factor in inducing fibrosis (76, 77). Firstly, under endogenous or exogenous stimulation, vascular lesions occur, and abnormal expression of vasoactive molecules changes vascular permeability, when endothelial cells are damaged or apoptosis and activated, recruiting inflammatory cells and immune cells (such as monocytes/macrophages, pDC) to the lesion site to cause inflammatory infiltration, and activating the innate immune response. These cells are activated to release pro-inflammatory and chemokines to induce tissue inflammatory response, microvascular damage and oxidative stress, which trigger fibrosis (74, 77), in which oxidative stress plays an important role. Abnormal oxidative stress has been found in SSc patients, that is, excessive production of ROS and an imbalance between oxidation and oxidation, and its effect with the inflammatory response (i.e., the effect of ROS on macrophage polarization and activation of inflammasome NLRP3 (78)) may promote the development of vascular lesions, and its induction effect on autoimmune disorders, endothelial dysfunction, and fibrosis is conducive to maintaining the pro-inflammatory state of SSc (79, 80).

In the adaptive immune process, T cells undergo inflammatory infiltration and abnormal expression, and partially differentiate into pathogenic T cells (such as Th17, Th1, TH2), which secrete pro-inflammatory factors to aggravate early tissue inflammation. Dysfunctional imbalances between pathogenic T cells and cells (Treg cells) which are beneficial to maintain immune homeostasis and abnormal differentiation of Treg cells might lead to SSc (77, 81). At the same time, IL-4 and IL-13, secreted by TH2 cells, exert a profibrotic role in SSc driving the deposition of ECM in fibroblasts, which is different from the disease-reversal effect shown in RA (81). B-cells activated by BAFF are stimulated by DC-presented antigens to produce autoantibodies that may have the ability to maintain and stimulate fibrosis of SSc (82), for example, the induction of persistent apoptosis of endothelial cells by endothelial autoantibodies (AECA) is beneficial to fibrotic lesions in SScs (83). B cells also secrete IL-6 to induce proliferative differentiation of autoreactive T cells and have pro-inflammatory effects, while direct or indirect contact between B cells and other cells is involved in the induction of fibrosis, cell activation and apoptosis, vascular lesions, and immune dysregulation processes (82). Moreover, T and B cells stimulate the proliferation, differentiation, and synthesis of fibroblasts in SScs by secreting cytokines (e.g., TNF-α, IL-6, TGF-β), promoting the progression of fibrosis (84, 85). This process leads to persistent inflammatory infiltration of SSc, which is linked to subsequent fibrosis and matrix deposition.

During the inflammatory phase, macrophages are activated to polarize into the M1 type, producing a large number of pro-inflammatory and chemokines, especially TGF-β (mainly produced by macrophages), which leads to pathological fibrosis. Early studies clearly showed that TGF-β is the most important effective inducer in fibrosis, stimulating the activation of fibroblasts and differentiation into myofibroblasts (the main effector cells for fibrosis formation), which could also be obtained through endothelial-mesenchymal transformation of endothelial cells. Myofibroblasts produce large amounts of collagen and express α-SAM, resulting in abnormal increase and excessive deposition of the extracellular matrix (ECM), which causes fibrosis (86, 87). The other effects of TGF-β on fibrosis have been described in other literatures, so they would not be repeated here. The occurrence of fibrosis and inflammation are inseparable, and chronic inflammation is one of the pathological features of SSc, so inflammation must play an important role in the pathogenesis of SSc. In addition to being the cause of fibrosis, its related pro-inflammatory factors and inflammatory mediators are also key participants in the pathogenesis of SSc. These pro-inflammatory factors not only act as promoters of inflammation, but also are a mediator to induce pathological fibrosis. For example, in SSc lung fibroblasts, inflammatory some NLRP3 mediated collagen synthesis by increasing miR-155 expression to promote fibrosis (88), while the effects of inflammatory factors IL-6, TNF-α, IL-4, and IL-13 on SSc have been discussed above. It can be seen that inflammation is an important player in the pathogenesis of SSc.

### 4.4 Sjogren syndrome

**Figure 4:**
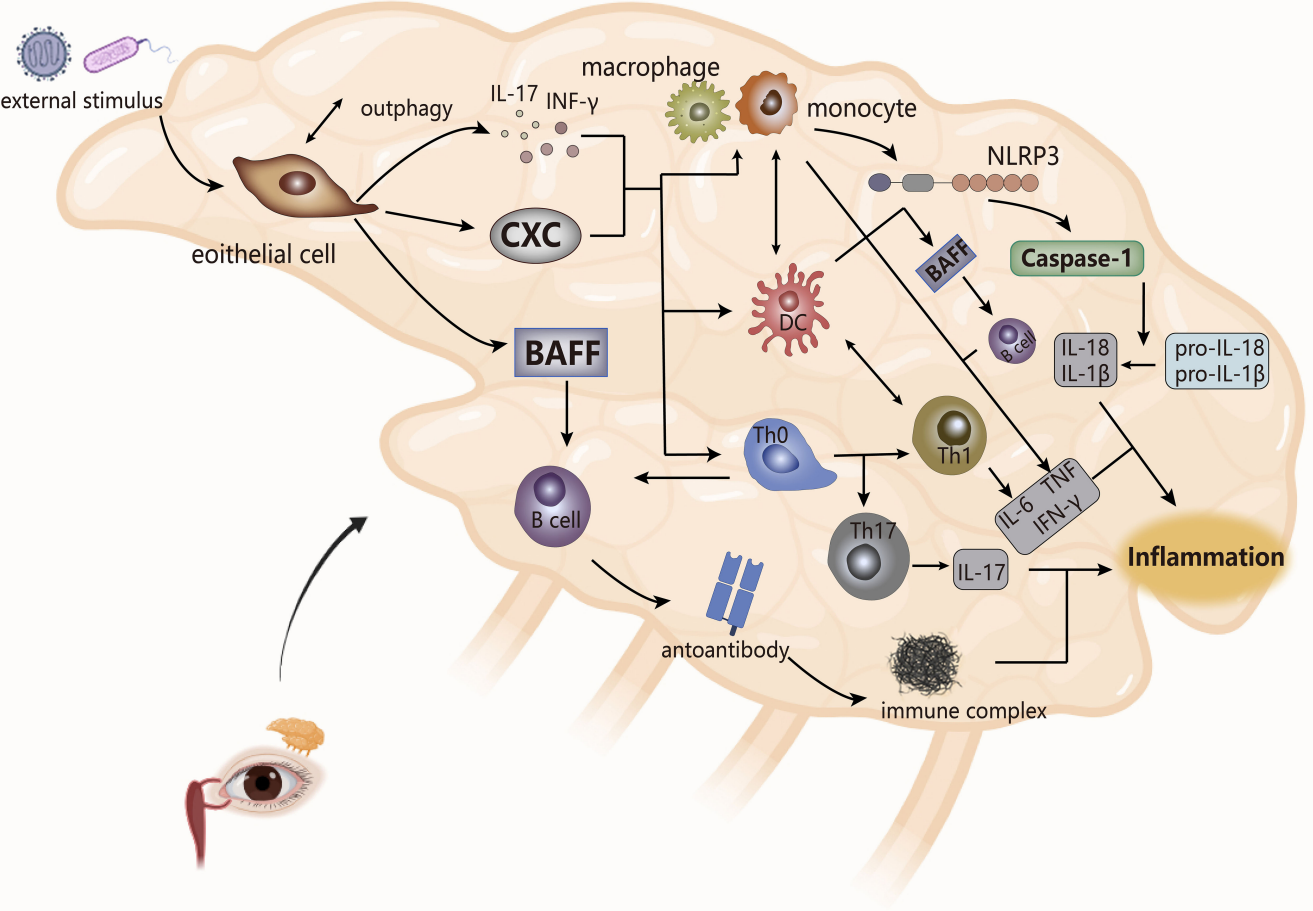
The pathogenesis of SS (take the lacrimal glands as an example). The cytokines produced by epithelial cells stimulate the secretion of inflammatory factors by monocytes and macrophages and the joint response of inflammasomes, which combine with the interaction between bound immune cells, ultimately leading to inflammation. NLRP3, inflammasomes. CXC, chemotaxis.

Sjogren syndrome (SS) (**Figure 4**) is a chronic systemic autoimmune disease, but it is more common in the lacrimal and salivary glands, and clinically manifests as keratoconjunctivitis sicca, dry mouth (89). SS is divided into primary Sjogren syndrome (pSS) and secondary Sjogren syndrome (sSS), in which sSS appears on the basis of other immune diseases (such as RA, SSc), so other AIDs have the possibility of sSS, and the symptom may overlap. The main pathological feature of SS is the dysfunction of the exocrine glands (mainly lacrimal and salivary glands), which is caused by the infiltration of the exocrine glands by immune cells (90).

Abnormal external stimuli triggers apoptosis or necrosis of cells in epithelial tissue and causes local inflammation of the gland, while Serena et al. showed that the induction of tissue inflammation of the gland to autophagy (anti-apoptotic pathway) of salivary gland epithelial cells derived the activation of these cells in inflammatory pSS (91). The activated epithelial cells secrete cytokines (pro-inflammatory factors, chemokines, BAFF), and upregulate the expression of adhesion factors to recruit immune cells (DC, lymphoid T and B cells) to the site of injury of the gland, which makes them abnormally activated (92, 93). T cells are the core players in the pathogenesis of SS, of which CD4 T lymphocytes account for the main (7). CD4 T cells are essentially immune regulation as a helper T cell. According to scRNA-Seq, studies have shown specific expansion of CD4 T cells in pSS patients, and the pathogenic effects of their cell subsets TFH, TH17, TH2 on SS have also been confirmed in multiple studies (94). In the lip salivary glands, high expression of TH2-related factors in infiltrating lymphocytes stimulated the formation of ectopic GC, which may be beneficial for infiltrating B cells to produce autoantibodies. Therefore, compared with its role in RA, TH2 may play a pathogenic effect in SS (95).

Stimulated by DC, T cells, and BAFF, B cells are overactivated. The hallmark event is the presence of ectopic germinal centers (GCs) in the glands of SS patients, followed by the production of autoantibodies that interfere with the expression of muscarinic receptors on the glands and the formation of immune complexes with ribonucleoproteins to worsen the infiltration process of immune cells to circulate the cycle of immune activation, eventually leading to tissue damage (89, 92, 96). At the same time, immune cells secrete cytokines (such as IL, TNF, MMPs) to damage the gland. IL and TNF, in addition to aggravating the local inflammation of the gland, destroy the release of acetylcholine to affect its effect on gland receptors, while MMPs interfere with the interaction between gland cells and cytoplasmic matrix, which leads to obstruction of gland secretion, thereby making gland dysfunction (92, 97).

The innate immune process of SS is accompanied by the infiltration of monocytes, and the presence of a large number of macrophages is detected. The number of monocytes is positively correlated with the level of tissue inflammation, and the resulting inflammation may drive the activation of epithelial cells, which affect the release of pro-inflammatory factors and the proliferation and differentiation of inflammatory cells, thereby maintaining the inflammatory state of the gland (98). Meanwhile, the activation of inflammasome NLRP3 and the upregulation of downstream caspase-1, IL-1β and IL-18 expression in infiltrating monocytes and macrophages were observed in patients with pSS. NLRP3 appeared to be activated by purinergic P2X7 receptors (P2X7R) and DNA deposits produced by persistent inflammatory conditions, and it ultimately mediated the pathogenesis of SS with the IFN pathway (99–101). Studies have also shown that in SS, angiogenesis was associated with gland inflammation. Neo angiogenesis accompanied by epithelial tissue lesion processes leaded to increased infiltration of monocytes, and the presence of vascular endothelial growth factor was detected in the inflammatory cells of the gland, so the formation of micro vessels may reflect the degree of chronic inflammatory lesions of gland tissue (102). At the same time, in the inflammatory microenvironment of SS patients, pro-inflammatory factors produced by inflammatory cells and immune cells form a complex cytokine network to intervene in the disease process. For instance, in AQP5-Cre mice, the upregulation of TNF-α expression weakened the immune dysfunction of the salivary glands and induced inflammation, accompanied by atrophy of acinar cells to reduce saliva secretion (103); In mouse models, IL-17 secreted by TH17 cells reduced saliva flow rate and aggravated glandular tissue damage (104); In SS, levels of IL-6 were associated with the amount of monocyte infiltration, inflammation of the salivary glands, and TH17 production (105).

In the pathogenesis of SS, inflammation may be an important element of salivary gland epithelial cell activation, and the concept of “autoimmune epitheliitis” has been proposed (106), and the interaction between this cell and innate, adaptive response leads to the occurrence of SS. In NZB/W F1 mice it has been verified that persistent inflammatory stimulation produced gland dysfunction, which was a catalyst for the development of SS-like diseases (107). At the same time, inflammation may be presented as a pathological feature of SS, including conjunctivitis, the complication interstitial pneumonia. Studies have shown that infiltration of inflammatory cells (macrophages) is not much associated with the degree of MSG lesion in SS patients, but may be related to adverse prognostic factors or later systemic features (108), which indirectly illustrates the expression of inflammation in the late stage of SS.

### 4.5 Ankylosing spondylitis

**Figure 5:**
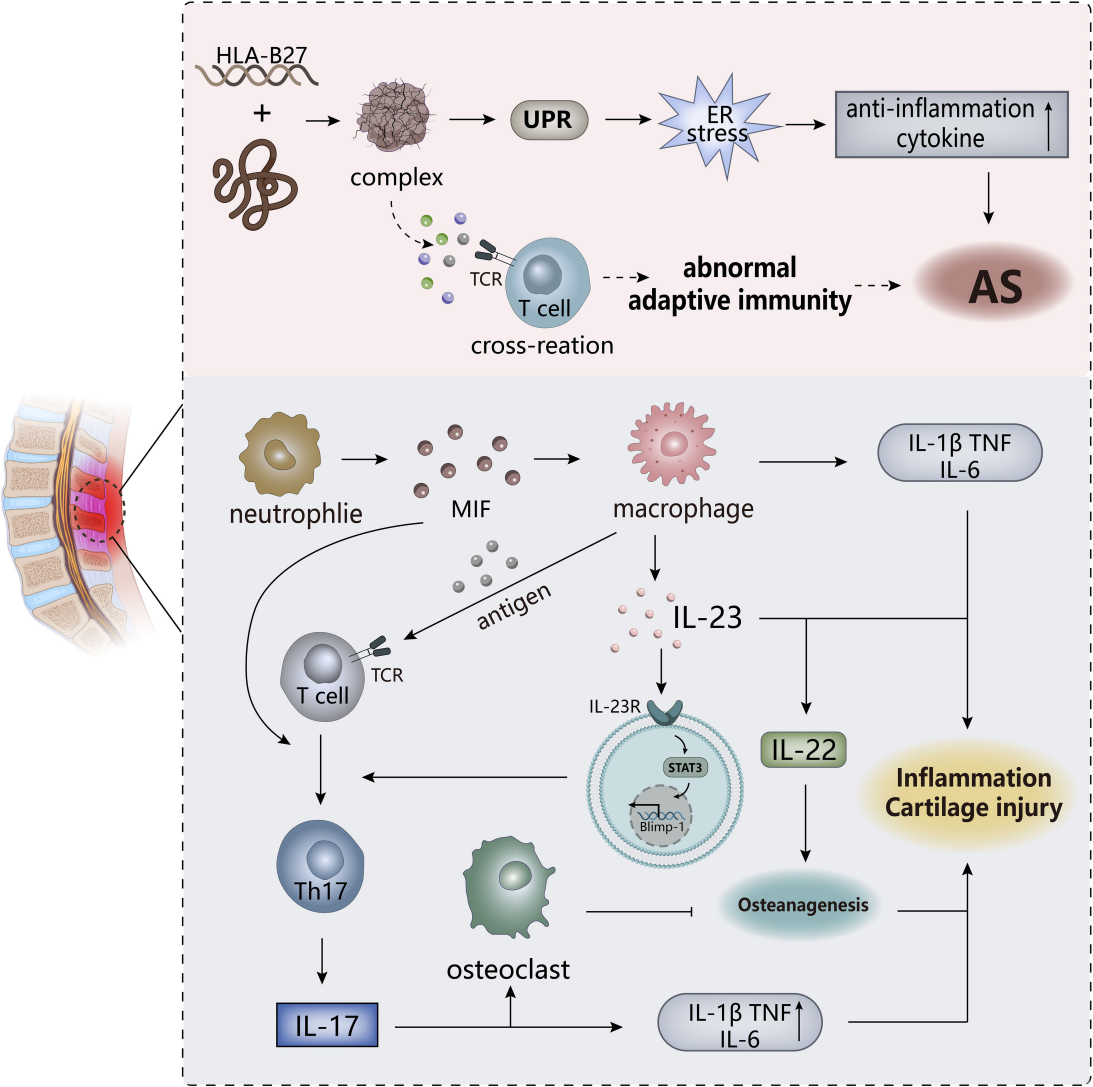
The pathogenesis of AS. ER stress triggered by the HLA-B27 gene may be the trigger for AS, and the T cell cross-reactivity triggered by the formed complex may also be one of the triggers. The IL-23/IL-17 pathway in the body plays an important role in the overall pathogenesis and is an important player in leading to inflammation and cartilage damage. MIF, macrophage migration inhibitor. TCR, T cell receptor.

Ankylosing spondylitis (AS) (**Figure 5**), also known as radiographic axial vertebral osteoarthritis (radiology axSpA), is a chronic systemic inflammatory rheumatoid disease. The main pathological features of AS are inflammation of spinal attachment points and sacroiliac joints, accompanied by inflammation of tendons and formation of ligamentous osteophytes, making it have osteogenic changes and osteolytic bone destruction, eventually leading to abnormal bony rigidity. AS is clinically manifested as arthritis, inflammatory back pain, spinal dysfunction that obstructs movement and extra-articular complications (109–111).

As an immune-mediated inflammatory disease, is AS classified as autoinflammatory or autoimmune disease? This point is not clearly defined. Both innate and adaptive immunity are involved in the pathogenesis of AS, among which human leukocyte antigen (HLA)-B27 has a strong correlation, with only about 20% genetic correlation, but may be an important predisposing factor of AS. There are related “articular peptide theory” and misfolded protein response hypothesis, the former is that HLA-B27 presented antigenic peptides trigger lymphocyte cross-reaction, and the abnormal adaptive immune response triggered is the basis for autoimmunity. The latter refers to the accumulation of incorrect or partial folds of HLA-B27 in the cell results in an endoplasmic reticulum (ER) stress response, which may lead to the activation of the unfolded protein response (UPR), subsequently triggering the activation of NF-κB. This induces the release of pro-inflammatory factors in nuclear cells/macrophages and promotes the development of AS inflammation, which indicate the inflammatory effects of HLA-B27 on AS (112, 113). The specific mechanisms of these two hypotheses have not been fully elucidated, and have not been fully combined, and are still partially questioned.

In addition to the importance of HLA-B27, the correlation between the IL-23/IL-17 pathway and AS has gradually been revealed by more experiments. IL-23 itself could drive attachment inflammation in spondyloarthropathy by binding to receptors affecting Rag-dependent cells (114), while ER stress induces macrophage polarization stimulation to produce IL-23 to upregulate the expression of transcription factor Blimp-1 through STAT3-dependence, inducing the differentiate of pathogenic helper T17(TH17) cells to develop an inflammatory cascade (115). TH17 cells specifically express the transcription factor ROR-γt to induce transcription of the IL-17 gene. Subsequently, the production of IL-17 promotes the secretion of IL-1, IL-6, TNF*-α* by other cells. IL-17 is synergistic with these factors to exert pro-inflammatory effects, ultimately inducing joint inflammation in AS (116). IL-17 could also stimulate the activation of osteoclasts to inhibit bone regeneration, but the downstream cytokine IL-22 of IL-23 has the effect of inducing osteoblast activation to stimulate bone proliferation (114, 117), so the IL-23/IL-17 pathway may explain the existence of two contradictory phenomena of bone erosion and new bone formation in AS patients. However, in another study, although it was confirmed that IL-23 and IL-17 expression were at an increased level and positively correlated in AS, it was shown that IL-23R-positive γ/δ T cells in peripheral blood secrete IL-17 to mediate the progression of AS, rather than TH17 cells, which may be related to the sample site of AS selected in the experiment (118), indicating that the sources of IL-17 are multifaceted, including neutrophils, macrophages, and innate lymphocytes.

Although both innate and adaptive immunity are involved in the pathogenesis of AS, according to the current research results, the innate immune system occupies a dominant position, in which innate immune cells (neutrophils, monocytes, macrophages, ILCs) play a key role. The role of macrophages in AS is mainly reflected in the secretion of a large number of pro-inflammatory factors (TNF-α, IL-1β and IL-23) after polarization and the stimulation of lymphoid T cell activation by antigen presentation (119). In inflamed tissues, macrophage migration inhibitor (MIF), mainly produced by neutrophils, acts as the upstream driver of pro-inflammatory factors and promotes the activation of TH17 cell-like phenotypes, which accelerate the emergence of SpA-like clinical features (120). Existing studies have shown that intestinal disorders have a strong correlation with the inflammation of AS. ILC3 in AS patients with intestinal inflammation migrated to peripheral blood, synovial fluid and bone marrow (BM) to expand after intestinal polarization to participate in the development of AS, as well as produce IL-17 and IL-22, in response to IL-23 to induce inflammation (121); In AS patients with intestinal inflammation, overexpression of NLRP3, NLRC4 and AIM2 were observed in inflammatory-infiltrating monocytes and epithelial cells, which might be driven by gut bacteria. And then, the inflammasome regulated IL-17, IL-22 and IL-1 expression through IL-23β induction, indirectly affecting the IL-23/IL-17 pathway. Intestinal dysbiosis may induce activation of innate immunity, and the resulting inflammasome activation may be involved in the formation of intestinal inflammation (122), so the effect of intestinal dysregulation on inflammation may be involved in the development of AS, which possibly has strongly associated with the ILC3 population of intestinal origin.

In addition to being a pathological feature of AS, inflammation is an important driver in the pathogenesis of AS. Early inflammation leaded to the destruction of intervertebral discs, followed by focal bone erosion and cartilage damage. This sustained destruction eventually contributed to excessive tissue formation and ectopic chondrocytes formation (123). The dysfunction of the inflammasome on the activation of autoreactive T cells and the effect of pro-inflammatory factors on bone hyperplasia have been confirmed (124), indicating that inflammation is an important cause of AS. In the late stage of AS, inflammation is more present as a pathological feature in various organs of the patient, such as arthritis, enthesitis and uveitis. One study showed that the occurrence of inflammation in the advanced stage of SpA was greatly correlated with IL-17, but situ analysis of IL-17 in the patient’s bone tissue samples showed that it was mainly produced by granulocytes, not TH17 cells (125), in which mast cells released stored exogenous IL-17A to amplify local tissue inflammation of peripheral SpA (126).Therefore, from the cytokine sources, innate immunity seems to be more involved in the pathogenesis of AS than adaptive immunity, and has a greater correlation with it.

In the adaptive immune response to AS, more research has shown that TH17 cell responses trigger inflammation in AS. In mouse models of SpA, the presented antigen activated cytotoxic CD8+ T cells, and immunodeficiency appeared to increase these immune responses, leading to the emergence of SpA-like diseases after combining genetic predisposition to dysfunction and autoreactivity of Treg cells (110). The role of B cells seems to be minimal in the pathogenesis of AS, and not many experiments have revealed it, but specific autoantibodies have been detected in serum samples of AS patients (127). Immune complexes, B cell activation and immune tolerance disruption all seem to be verified (110), making AS seemly have some characteristics of autoimmunity and as AIDs possibly. Although more evidence suggests that AS is more likely to be a chronic autoinflammatory disease, autoimmunity and autoinflammation seem to be connected in the pathogenesis of AS, but a clear dominance of one may help the study of the treatment strategy of this disease. The autoimmune characteristics in AS should be explored later, such as the role of autoantibodies, which is conducive to a clearer elucidating of the pathogenesis of AS.

### 4.6 Autoimmune hepatitis

**Figure 6:**
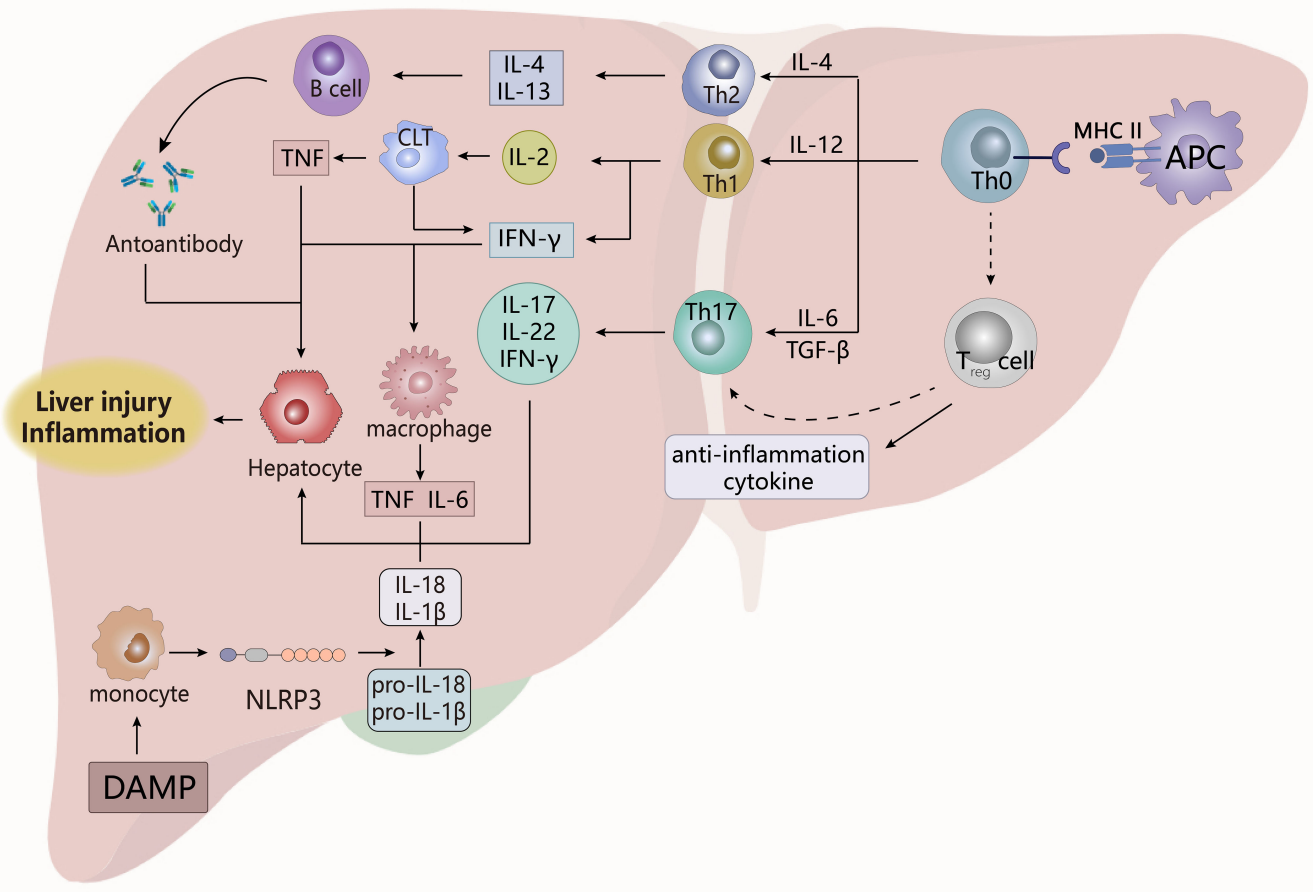
The pathogenesis of AIH. The activation of T cells by antigen-presenting cells prompts them to differentiate into multiple cells, which act in tandem with B cells and innate immune cells by secreting different inflammatory factors to promote apoptosis and damage of liver cells, which eventually leads to the appearance of chronic hepatitis. Treg cells, regulatory T cells; DAMP, damage-related molecular patterns. APCs, antigen-presenting cells. CLT, Cytotoxic lymphoid T cells.

Autoimmune hepatitis (AIH) (**Figure 6**), one of the most common autoimmune liver diseases (AILD), is a persistent inflammatory disease with women as the main affected population. The main pathological features of AIH are interface hepatitis, autoantibodies and lymphocyte infiltration, but there is no significant specific clinical phenotype, making diagnosis very difficult, and later inflammation, liver fibrosis and liver failure would occur (128, 129).

The core key to the pathogenesis of AIH is the destruction of the liver’s immune tolerance, which triggers the imbalance between effector cells and Treg cells in the liver. This results in the liver’s immune response to autoantigens, eventually leading to autoreactive liver damage and continuous occurrence (129), and the liver dysfunction leads to liver failure. Regulation of T cells plays a key role in the pathogenic process of AIH. After the resting antigen-presenting cells (APCs) are activated, the autoantigen peptide is presented to the naïve T cells (TH0) through the T cell receptor (TCR), so that TH0 is differentiated into TH1, TH2 and TH17, and recruited to the site of liver injury (130, 131). In the liver, a variety of cells could act as APCs, including dendritic cells, Kupfey cells, hepatic sinus endothelial cells, hepatic stellate cells (132). TH1, TH2 and TH17 exert pathogenic effects, TH2 secretes IL-4, IL-10, 1L-13 to promote the maturation of B cells, thereby promoting the production of autoantibodies (130, 131), such as ANA, anti-smooth muscle antibodies (SMA), anti-liver and kidney microsomal type 1 (anti-LKM1) antibodies, anti-LKM3 antibodies and anti-hepatic cytoplasmic type 1 (anti-LC1) antibodies, these autoantibodies bind to hepatocytes to cause toxic reactions (130); IL-2 produced by TH1 induces the expression of HLA class I molecules on cytotoxic T cells, while the resulting IFN-γ provokes the expression of HLA molecules on hepatocytes, and ultimately stimulates effector T cells to trigger adaptive autoimmunity. TH17 secretes IL-17 and IL-22 to affect hepatocyte damage and tissue inflammation (130, 131), and the above process eventually leads to liver parenchymal damage and worsening of inflammation.

In AIH, Treg cell defects are advantageous for the maintenance of autoimmune responses and loss of immune tolerance. Stimulated by TGF-β, TH0 differentiates Treg cells to produce anti-inflammatory factors that play a role in maintaining immune homeostasis (130). Treg cells themselves are functionally repaired in immunoregulation, and although previous experiments have confirmed that the number and proliferation of CD4+CD25+Treg cells are reduced in active disease (133, 134). More studies have shown that the number and expansion of functional CD4+CD25+FOXP3+Treg cells are increasing in AIH patients. The frequency of Treg cells increases with the degree of inflammation in patients, and they migrate and accumulate to inflamed parts of the liver, which appears to be associated with stimulation of the inflammatory factors TGF-β, IL-2, and the chemokine CXCR3/CXCL9 (135–137). This contradictory result seems to have population differences with a phenomenon of patient heterogeneity, that is, the number of Treg cells decrease in pediatric patients but enrich in the adult’s liver. This is possible because the child’s development is not mature enough, so Treg cells are not very resistant to the effects of external adverse stimuli. The difference in the selection criteria for Treg cells in the previous and later studies might make the final conclusion different, which needs to be confirmed by further experiments. However, immune system disorders in the AIH have greatly increased apoptosis in Treg cells (138), although whether this affects Treg cells’ control of the disease has not been experimentally explained.

However, Treg cells may undergo pathogenic transitions in AIH. Arterbery et al. found that newly onset AIH patients had the transformation of FOXP3 Treg cells to a pro-inflammatory phenotype, that was, an increase in the frequency of TH1-like Treg cells and TH17-like Treg cells. Subsequently, secretion of effector factors IL-17 and IFN-γ were important participants in the pathogenesis of AIH. This transition seemed to be related to the negative impact of the inflammatory microenvironment on Treg cells. Inflammatory factors secreted by monocytes (such as IL-12 and IL-6) might promote the pro-inflammatory phenotype of Treg cells to make it malfunction, eventually inducing persistent chronic inflammatory states (139); Toll-like receptors on CD14 monocytes were stimulated by damage-related molecular patterns (DAMP) to activate inflammasome NLRP3 and its signaling pathway, so that the secretion of IL-12, IL-1β and IL-18 was enhanced. Under the action of inflammatory factors, Treg cells transformed to a pro-inflammatory phenotype to cause dysfunction, thereby promoting autoimmunity, while monocytes also stimulated apoptosis of hepatocytes to aggravate hepatitis (140). Therefore, the regulation of Treg cells by the pro-inflammatory environment may be a key factor in the pathogenesis of emerging AIH. In the ConA-induced AIH mouse model, it has shown that pathogenic NLRP3 had a promoting effect on liver injury and hepatitis, which might be activated by ROS produced by inflammatory cells in the inflamed site. Subsequently it stimulated caspase-1-mediated pyroptosis and IL-1β production to aggravate damage and inflammation in AIH (141). In addition, the infiltration of inflammatory cells in AIH patients is observed, which play an important role in maintaining the state of hepatitis through a large amount of pro-inflammatory factors. Macrophages can be stimulated by IFN-γ secreted by TH2 to produce IL-1, TNF-α (130), while monocytes are activated to spontaneously migrate to the site of liver injury to aggravate the degree of inflammation. The over-activation of monocytes seems to be enhanced by conventional Treg cells (142). Therefore, in addition to directly accelerating liver injury and inflammation, inflammatory mediators could mediate the action of T cells in autoimmunity to influence the pathogenesis of AIH.

### 4.7 Inflammatory bowel diseases

**Figure 7:**
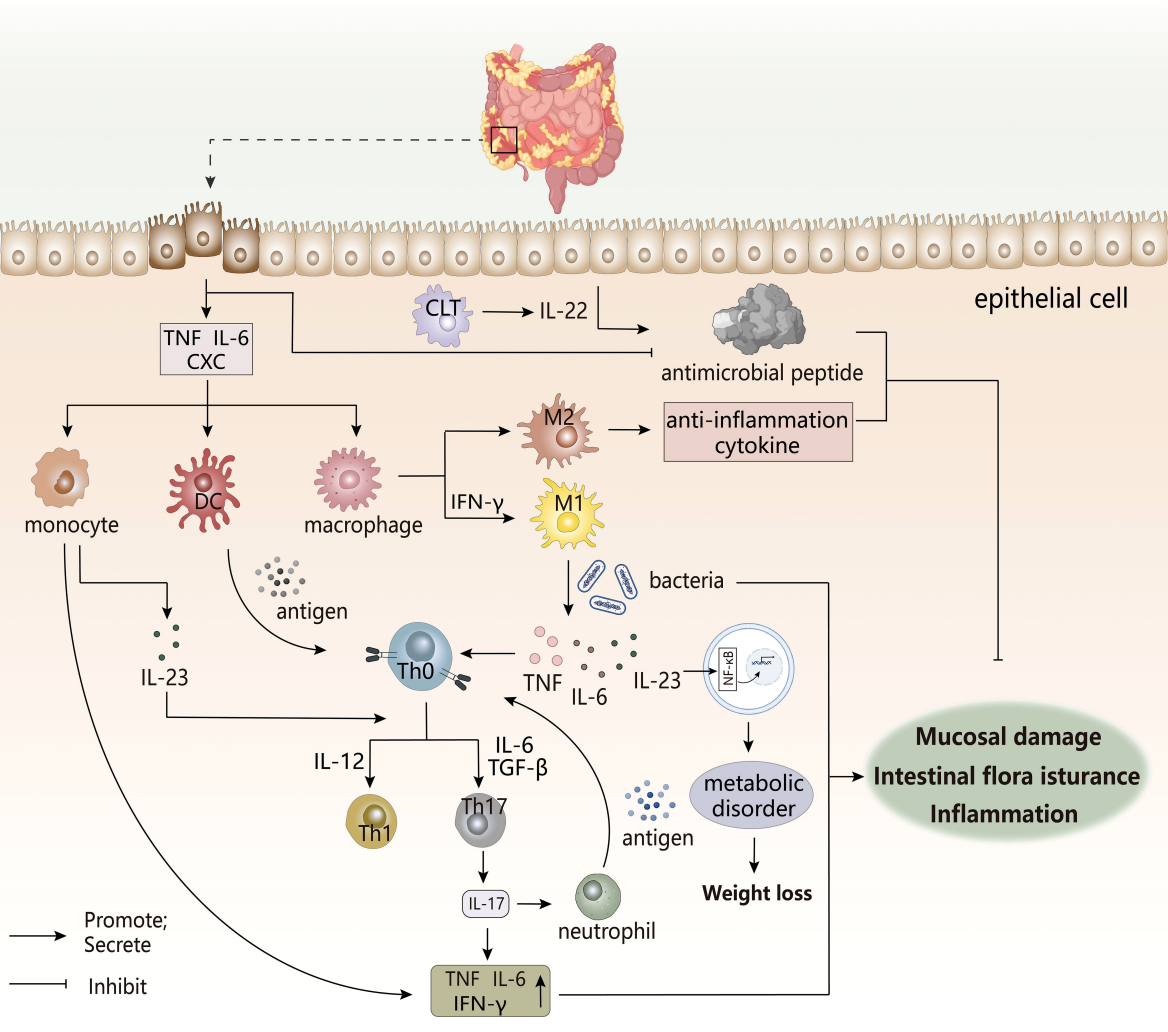
The pathogenesis of IBDs. The occurrence of IBD is often accompanied by immune disorders, intestinal flora disorders, and metabolic disorders, which are related to the imbalance regulation between inflammatory cells, immune cells, and intestinal groups. The damage of this intestinal epithelial cell triggers a cascading response by the immune system, and the various cytokines produced can trigger the destruction of the intestinal mucosal barrier, leading to the appearance of intestinal inflammation.CLT, Cytotoxic lymphoid T cells. CXC, chemotaxis.

Inflammatory bowel diseases (IBDs) (**Figure 7**) are an inflammatory autoimmune disease characterized by chronic intestinal inflammation, mainly including Crohn’s disease (CD) and ulcerative colitis (UC). The clinical manifestations of IBDs involve abdominal pain, diarrhea, blood in the stool, and weight loss. UC usually occurs only in the colon and rectal mucosa, while CD possibly occurs in all parts of the gastrointestinal tract (143, 144). The appearance of IBDs may have the following causes: impaired mucosal barrier, intestinal flora infection, immune dysregulation, intestinal dysbiosis (145). The root cause of IBDs may be a disorder of the mucosal immune system, which may be triggered by damaged intestinal epithelial cells or abnormal intestinal flora. The dysregulated mucosal immune system produces an excessive immune response to the normal microbial composition of the intestine to lead to the destruction of intestinal immune tolerance, thereby inducing intestinal inflammation (146).

The pro-inflammatory factors and chemokines secreted by intestinal epithelial cells after injury promote the infiltration of innate immune cells to the inflammatory site (147), while the antimicrobial peptides produced by epithelial cells that have a protective effect on the body are reduced in IBDs patients. This increases the enrichment of immune cells and the translocation of intestinal flora, thereby inducing inflammation, in which defensins have antibacterial properties. However, in UC patients, under the inducement of the pro-inflammatory factor TNF-α, IL-6, the abnormal increasement of HBD-2 of Defensin-β may exacerbate the inflammatory response (148). Under normal conditions, M2 macrophages secrete anti-inflammatory factors to maintain immune homeostasis, but once overstimulated, there would be a tendency to polarize to M1 type (147). Local inflammatory microenvironment of the intestine might stimulate the transformation of macrophages to pro-inflammatory phenotypes. Studies have shown that there is a unique subset of intestinal macrophages CD14 in the inflamed mucosa of IBDs patients, which are obtained by abnormal differentiation of macrophages induced by IFN-γ. Under the action of coexisting bacteria, excess IL-23, TNF-α, and IL-6 produced by macrophages may induce effector T cells to produce IFN-γ in response to TH17/TH1 cell responses. The forming IL-23/IFN-γ axis affects local inflammation in the gut, while the role of IL-23/IL-17 axis may be more manifested in systemic inflammation (149). In addition, macrophages can also stimulate TH17/TH1 differentiation as antigen-presenting cells. DC cells undergo antigen presentation to activate the immune response of T cells. Next, IL-23 secreted by these two cells after activation not only participates T cell differentiation, but also stimulates ILCs to produce IL-22 to promote epithelial cells to produce antimicrobial peptides to maintain intestinal homeostasis, but dysfunction of ILCs may still adversely affect IBDs (147). So, these innate immune cells are key initiators or continuators of IBD.

The pathogenic TH17 immune response appears to dominate the pathogenesis of IBD, performing key pro-inflammatory processes. TH17-related cytokines seemingly have increased expression at the site of inflammation in IBD patients, in which IL-17 expression in the inflamed mucosa of active CD and UC patients was significantly increased, mainly derived from monocytes/macrophages and T cells (148). IL-17 would aggravate the induction and persistence of inflammation by increasing the expression and production of pro-inflammatory factors. The differentiation of IL-17-producing T cells can be induced by TGF-β stimulated by IL-6, while the inhibitory effect of TGF-β on TH1 and TH2 differentiation indicates its anti-inflammatory potential, and this difference appears to be related to systemic or local expression (150). However, studies have shown that TH17 cells induced by TGF-β and IL-6 appear to be non-pathogenic, while IL-23/IL-6/IL-1β stimulation produces inflammatory TH17 cell phenotype (151), which indirectly explains the disease-promoting effect of the IL-23/IL-17 axis. Th17 cells also indirectly promote the migration and recruitment of neutrophils by secreting IL-17 to induce other factors, while neutrophils in turn present antigens to stimulate T cells (152), thereby maintaining the occurrence of intestinal inflammation.

A healthy gut microbiota is an important factor in maintaining homeostasis, but the presence of ecological disturbance in the co-existing flora could have an adverse effect and increase the burden of chronic inflammation. In IBDs patients, the number of anti-inflammatory properties (eg, Bifidobacteria) decreases, while pathogenic adherent Escherichia coli has an abnormal increase (153), which adheres to and invade intestinal epithelial cells (154). Its continuation of intestinal inflammation may be achieved by mediating the differentiation of TH17 cells, the development of Treg cells, promotes the differentiation and recruitment of inflammatory cells, and stimulates the release of their pro-inflammatory factors, ultimately resulting in the presence of more pathogenic T cells in the gut (155, 156). At the same time, deficiencies of inflammasome NLRP6 in gut may alter the ecological regulation of the fecal microbiome to drive the onset and worsening of intestinal inflammation (157). Studies have also shown a correlation between malnutrition, intestinal flora and intestinal inflammation. A stronger response to inflammation was observed in nutrient-deficient individuals, which might aggravate the inflammatory response to produce more pro-inflammatory factors, ultimately leading to systemic chronic inflammation. Intestinal inflammation and impaired mucosal barriers might lead to bacterial translocations to alter gut microbial composition, which influenced the metabolism and absorption of nutrients (158). In the meantime, inflammatory factors reduced the synthesis of metabolic hormones by activating NF-κB, and affected appetite, which was not conducive to the body’s absorption of nutrients, aggravating the body’s malnutrition (159). This whole process is the result of a vicious circle, which also explains the clinical characteristics of weight loss in IBDs patients.

## 5 Targeted therapy related to inflammation

**Figure 8:**
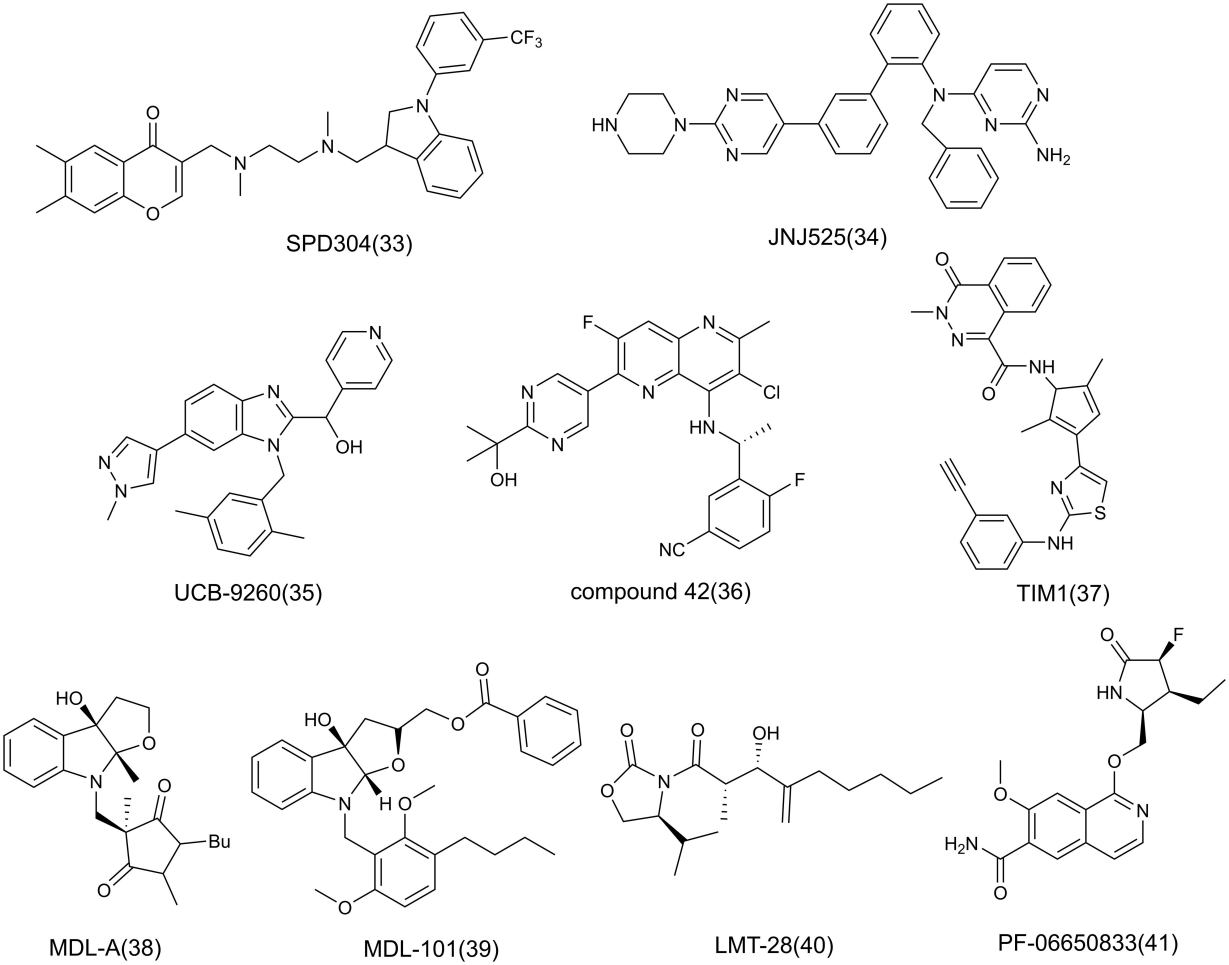
The structure of small molecule inhibitors.

At present, the treatment of AIDs focuses on the use of immunosuppressants, and the application of anti-inflammatory strategies in AIDs is gradually increasing. The following will focus on the role of key inflammatory factors or mediators in the pathogenesis, and briefly introduce the inhibitors of many popular targets such as TNF-α, IL-6, IL-1 in inflammation, mainly including antibodies (**Table 1**), small molecule compounds (**Figure 8**), and natural products related to the corresponding targets, and antibodies are the main ones, in which antibodies are classified based on targets.

**Table 1:** The antibodies that could be used to treat AIDs.

Class	Name	Type	Target	Major indication	Research stage
TNF inhibitor	Infliximab	mAb	TNF-α	RA,CD,UC,AS,pasoriasis	marketed
	Etanercept	fusion protein	TNF	AS, psoriasis, juvenile idiopathic arthritis, RA	marketed
	Adalimumab	mAb	TNF	RA, AS, CD, UC, psoriasis	marketed
	Certolizumab pegol	mAb	TNF	CD, RA, AS	marketed
	Golimumab	mAb	TNF	RA, psoriatic arthritis, AS, UC	marketed
	Ozoralizumab	nanobody	TNF-α	RA	marketed
IL-6 inhibitor	Tocilizumab	mAb	IL-6R	RA, juvenile idiopathic arthritis, CD, SLE, SSc	marketed
	Sarilumab	mAb	IL-6R	RA	marketed
	Sirukumab	mAb	IL-6	RA, SLE	phase III
	Clazakizumab	mAb	IL-6	RA	phase III
	Olokizumab	mAb	IL-6	RA	phase III
	ALX-0061	nanobody	IL-6R, HAS	RA	phase II
IL-1 inhibitor	Anakinra	recombinant IL-1Ra	IL-1R	RA, NOMID	marketed
	Canakinumab	mAb	IL-1β	CAPSs, sJIA	marketed
	Rilonacept	fusion protein	IL-1β	recurrent pericarditis, CAPSs, sJIA	marketed
	Gevokizumab	mAb	IL-1β	autoinflammatory diseases	marketed
IL-17 inhibitor	Secukinumab	mAb	IL-17A	PsA, AS, psoriasis, PSO, axSpA	marketed
	Ixekizumab	mAb	IL-17A or IL-17A/F	PsA, AS, psoriasis, PSO, axSpA	marketed
	Brodaluma	mAb	IL-17R	Psoriasis, PsA	marketed
	Bimekizuma	mAb	IL-17A, IL-17F	PSO, PsA, AS, axSpA	marketed
	Sonelokimab	nanobody	IL-17A/F	PsA, PSO	phase II
	Netakimab	mAb	IL-17A	PSO, AS	phase III
	Vunakizumab	mAb	IL-17A	PSO	phase II
	CNTO6785	mAb	IL-17A	RA	phase II
	COVA322	FynomAb	TNF, IL-17A	PsA	phase I
	ABT-122	mutant immunoglobulin (DVD-Ig™) molecule	TNF, IL-17A	RA, PsA	phase II
	CJM112	mAb	IL-17A	PSO	phase I
IL-12/23 inhibitor	Ustekinumab	mAb	IL-12/23 p40	CD, UC, PsA, PSO	marketed
	Guselkumab	mAb	IL-23 p19	PsA, PSO, IBD	marketed
	Tildrakizumab	mAb	IL-23 p19	PSO, PsA	marketed
	Risankizumab	mAb	IL-23	PSO, PsA, CD	marketed
	Mirikizumab	mAb	IL-23 p19	UC	marketed

### 5.1 Antibodies

#### 5.1.1 TNF-α

##### 5.1.1.1 Infliximab and its biosimilars (1)

Infliximab(IFX; Remicade™), a chimeric IgG1-infused human monoclonal antibody (mAb) that selectively blocks TNF-α activity, was developed early and was the first biologic agent approved for the treatment of CD and UC (160). IFX is now approved in various countries for monotherapy or combined administration, and is widely used to treat moderate and severe RA, active AS, and psoriasis (161), which might have better relief when used early in the disease. However, IFX undergoes loss of response over time and serious adverse effects, such as infection, infusion reactions, hypersensitivity reactions (160), which lead to greatly reduced patient compliance, so when treating these immune-mediated inflammatory diseases, active therapeutic drug monitoring (TDM) of IFX leads to better outcomes (162).

Biosimilars of IFX are currently approved by the FDA and EMA for the treatment of IBD, including PF-66438179, CT-P13, SB-2, ABP 710 (163). CT-P13 (Remsima) is the first developed IFX biosimilar, produced in the same cell line (an SP2/0 murine cell line), with the same amino acid sequence, and the efficacy, safety, and immunogenicity in most clinical trials are comparable to IFX (163). While CT-P13 is commonly administered intravenously, subcutaneous CT-P13 has been developed and shown to have a similar safety and durability to intravenous treatment for IBD (NCT02148640), resulting in higher patient satisfaction and acceptance (164). This route of administration shift not only improves patient compliance but may also help reduce the risk of infection. Moreover, a 13-year global study of CT-P13 after its launch is ongoing (NCT02557295) (165). SB-2 differs from Infliximab in that the murine cells used (a Chinese hamster ovary (CHO) cell line) are different, which may lead to differences in C-terminal Lys residues, changing the proportion of their different charge isomers, but this does not affect the site recognition and antigen fragment binding of SB-2, which has a high degree of biological similarity with IFX. SB-2 is currently approved by the European Union in 2015 for the treatment of IFX indications (163, 166). PF-06438179 (GP1111) is another IFX biosimilar produced after SB-2, developed in accordance with regulatory recommendations from the FDA and EMA. PF-06438179 has differences in N-glycosylation and charge heterogeneity caused by C-terminal Lys compared to IFX, but these have no clinical relevance. It has been approved for the treatment of the indications used for IFX, and all evidence indicates the biosimilarity between PF-06438179 and IFX (167). ABP 710 (AVSOLA) is developed as an IFX reference product using the same CHO cell line as SB-2 and has been approved for clinical treatment in the United States and Canada (168). Therefore, a large body of evidence suggests that there is no clinically significant difference between IFX and its biosimilars, and that drug translation does not increase the risk of disease in patients, indicating the safety and efficacy of biosimilars.

##### 5.1.1.2 Etanercept and its biosimilars (2)

Etanercept is a dimeric fusion protein produced by recombinant DNA consisting of the extracellular fraction of human p75 TNFR with the Fc fraction of IgG1, the presence of the latter fraction giving it a half-life of up to 4.8 days. As an inhibitor, Etanercept competitively binds to soluble and membrane-bound TNF to inhibit its activity, exhibiting high binding capacity (Ki = 10^-10^M), which has effective relief of inflammation, and now is used to treat severely active AS, psoriasis, juvenile idiopathic arthritis, especially moderately or severely active RA (169). Because Etanercept is administered subcutaneously, the most common adverse reactions are injection site reaction (ISR) and serious infection, but it combined with methotrexate (MTX) may reduce the incidence of ISR and shows better clinical efficacy in the treatment of RA (170). Due to the high production cost of Etanercept, leading to only a small number of people have the affordable availability, the emergence of biosimilars is very necessary. Some biosimilars of Etanercept have been developed, including SB4, GP2015, LBEC0101, DWP422, HD203, CHS-0214, TuNEX/ENIA11^®^. These antibodies have been incorporated into clinical use and have shown better efficacy in the treatment of RA patients who do not respond to MTX, even better than Etanercept, but there is a lack of real-world research data to confirm (171).

##### 5.1.1.3 Adalimumab and its biosimilars (3)

Adalimumab (Humira^®^, AbbVie) is a fully human IgG1 mAb obtained by bacteriophage display technology, consisting of two κ light chains and one heavy chain, with a total molecular weight of 148 kDa, which only binds specifically to soluble TNF (Kd = 6×10^-10^ M) (172, 173). Adalimumab was approved for clinical use by the EMA in 2003, initially primarily for the treatment of RA, and is now also used for the treatment of AS, CD, UC and psoriasis, and the indications are increasing all the time (173). Compared to other TNFi, Adalimumab appears to have the broadest range of therapeutic indications. However, the cost of using Adalimumab is relatively high, imposes a significant financial burden on patients, which may limit its use. With the time of administration, the proportion of patients with anti-drug antibodies increases, so biosimilars are gradually being developed. There are currently more than ten biosimilars, including ABP 501, SB5, FKS327, BI695501, MSB11022, GP2017, PF-061410293, CTP17, AV702. Most of these antibodies are composed of two heavy chains and two light chains of the κ subclass, with molecular weights comparable to Adalimumab. According to a large number of preclinical studies and clinical trial results, these antibodies have a high degree of similar clinical efficacy to Adalimumab with no significant difference, and the immunogenicity of it is also comparable, which have generally been used in the treatment of related indications (173, 174).

##### 5.1.1.4 Certolizumab pegol (4)

Certolizumab pegol (Cimzia) is a humanized mAb of recombinant and polydiethanolated Fab’ fragments, in which the fragments of Fab are synthesized by microbial fermentation in Escherichia^®^ coli through DNA recombinant technology. It has the advantages of low cost, short cycle, good returns and sufficient sources (175). From the structural point, Certolizumab lacks the Fc part of IgG1 in previous anti-TNF drugs, resulting in different performance in *in vitro* experiments. The structural modification of polydiethanolation may be beneficial to its half-life, penetration and staying power *in vivo*, ultimately resulting in Certolizumab specifically binds to TNF-α, which is used to treat CD, RA, AS (176). Certolizumab, whether alone or in combination with MTX, has been shown to be good at alleviating clinical signs and reducing joint damage in patients with RA. The efficacy of Certolizumab appears to be comparable to other TNF-α inhibitors, due to its structural differences may have lower immunogenicity (6.9%), which helps reduce the risk of infusion reactions and allergic reactions in patients (177). However, Certolizumab appears to have a greater risk of serious infection and a higher frequency of adverse events (NCT01491815), but lack of clinical data related to safety in long-term use (178), so the duration of treatment needs to be confirmed when choosing Certolizumab.

##### 5.1.1.5 Golimumab (5)

Golimumab (Simponi™) is a mAb of human immunoglobulin G1κ produced by knocking human immunoglobulin genes into the mouse genome, with subcutaneous and intravenous injection, only once a month, which is now used to treat multiple inflammatory AIDs, such as RA, psoriatic arthritis, AS, UC (179). The main feature of Golimumab is that it has high binding to TNF-α and low immunogenicity, and its binding force to soluble TNF-α is 19 pM measured by surface plasmon resonance, which seems to be comparable to Etanercept, but significantly higher than other antibodies. The immunogenicity is the lowest (3.8%) (compared with the above inhibitors), which indicates that the proportion of patients with anti-drug antibodies is the smallest, so it is not likely to occur hypersensitivity reactions, infusion response and has easier drug efficacy (180). Although these adverse effects are a few, the frequency of infection is increased in patients with Golimumab, particularly the risk of tuberculosis, so screening or potential viral testing is necessary before patients receive treatment to reduce the frequency of infection (179, 180).

##### 5.1.1.6 Ozoralizumab (6)

Ozoralizumab is a 38-kd trivalent anti-TNFα NANOBODY compound consisting of two humanized anti-human TNF VHH antibodies and one humanized anti-human serum albumin (HSA) VHH antibody. The presence of the latter part gives it a long half-life, namely, there is t_1/2_ for 30 days after subcutaneous injection of 30mg, which has been approved by Japan for the treatment of RA in 2022 (181). Ozoralizumab had the potent inhibition to arthritis and showed low immunogenicity and long-term efficacy, which were demonstrated in transgenic mouse models, possibly due to the special structural composition of Ozoralizumab. It seemed to tend to form small immune complexes (ICs) with TNF-α trimers that were not easily recognized by Fcγ receptors on immune cells to trigger additional immune responses. Therefore, in animal models, ICs were not easy to induce neutrophil recruitment at the injection site to stimulate acute inflammation, so the frequency of ISR was low, which indirectly indicated that Ozoralizumab was an effective candidate for alleviating inflammation (182, 183). In a phase II/III trial (NCT01007175) of Ozoralizumab in combination with MTX in the treatment of RA, patients experienced improvements in signs and symptoms with acceptable safety and tolerability (184). Therefore, Ozoralizumab is expected to be subsequently marketed in other countries for the treatment of RA.

#### 5.1.2 IL-6

##### 5.1.2.1 Tocilizumab (7)

Tocilizumab (TCZ; MRA) is the first humanized mAb that blocks IL-6 by transplanting the complementarity determining region of the anti-human IL-6 receptor of mice into human IgG1 using genetically engineered recombinant technology. TCZ competitively binds specifically to IL-6R to inhibit IL-6 activity, and is used to treat active RA, juvenile idiopathic arthritis, CD, SLE, and SSc. As a drug for RA, TCZ has the advantages of low immunogenicity, long-term and short-term efficacy, and good monotherapy, but the risk of infection is still relatively high (185, 186). In recent years, TCZ has been increasingly studied for the treatment of COVID-19 and appears to be effective in reducing mortality, and other indications (giant cell arteritis, polymyalgia rheumatica) are increasing.

##### 5.1.2.2 Sarilumab (8)

Sarilumab (REGN88; Kevzara^®^), a human IgG1 mAb, was approved by the FDA in 2017 for the treatment of RA. Compared with TCZ, it appears to have a higher affinity for the target, with Kd of 61.9 pM and 12.8 pM for recombinant monomer and dimer hIL-6Rα, respectively, and has higher inhibitory efficacy against IL-6, but no significant difference in safety has been observed in clinical trials (NCT01768572) (187, 188). Whether alone or in combination therapy, Sarilumab seems to have a good therapeutic effect on RA and improvement of physical function, and its efficacy is higher than Adalimumab. In patients with insufficient response to antirheumatic drugs, inflammatory symptoms and cartilage damage in patients treated with Sarilumab are significantly relieved. ADA in a small number of patients seems to have no effect on its efficacy and adverse effects, and might have greater advantages for the treatment of RA. However, when choosing combination therapy, Sarilumab is more likely to be used in combination with conventionally synthesized DMARDs, and it binding to biological DMARDs appears to increase the risk of immunosuppression and infection (189). Overall, Sarilumab has good therapeutic prospects as the second IL-6 inhibitor on the market, which is now also beginning to be used as a treatment for COVID-19.

##### 5.1.2.3 Sirukumab (9)

Sirukumab(SRK; CNTO 136) is a human IgG1κ mAb against IL-6 that specifically targets soluble IL-6 to block signaling of STAT3 (190). Based on phase I and II trials, phase III clinical trials (NCT01604343, NCT01606761, NCT02019472, NCT01689532, NCT01856309) for inflammatory diseases (RA, SLE) have been completed, and SRK produces therapeutic effects and adverse reactions that are comparable to other IL-6 blockers, showing a longer half-life (>15 days) and decreased levels of CRP (a nonspecific marker of inflammation activation), and a few ADA occurred. However, it was not known whether it was associated with a reduction in CRP due to higher mortality from severe infection and cardiovascular disease in later follow-up (191). In the latest clinical trial (NCT01856309), SRK maintained the safety and efficacy consistent with previous trials, namely the reduction in symptom of RA and improvement in physical function (192). Therefore, more clinical trials are needed to confirm whether SRK is truly incorporated into clinical use.

##### 5.1.2.4 Clazakizumab (10)

Clazakizumab (CLZ; BMS945429; ALD518) is a humanized mAb that specifically targets IL-6 using rabbit antibodies, with a high affinity of about 4 pM for human IL-6, which produced in Pichia pastoris yeast, and has entered clinical stage (193). In multiple phase II trials (NCT01373151, NCT00867516) with moderate or severe RA or inadequate response to MTX, CLZ had a long half-life, was well tolerated, had rapid improvements in patient mobility and HRQoL, and changes in laboratory indicators (eg, increased aminotransferases, slight injection responses, neutrophil reductions) were within the range of IL-6 inhibitors. Its combination therapy with MTX has shown better efficacy than monotherapy (194, 195) and has also demonstrated good tolerability and safety of CLA in a phase IIb trial (NCT01490450) with active psoriatic arthritis (196), and a clinical trial on CLZ therapy in patients who do not respond adequately to TNF inhibitors (NCT02015520) has been completed, but results have not yet been disclosed. Clinical trials of CLZ for other indications for non-AID are ongoing, suggesting that CLZ has great potential for inclusion in clinical use.

##### 5.1.2.5 Olokizumab (11)

Olokizumab)OKZ; CDP 6038), an anti-IL-6 mAb obtained after humanization of antibody 132E09 produced in immune rats, exerts inhibitory activity by binding to IL-6 at site3 (Kd = 10 pM) to block the gp130 signaling that forms hexamers, showing significant anti-inflammatory effects in arthritis models (197), which is currently in the clinical stage of the treatment of RA. According to previous studies, OKZ has a half-life of up to 31 days, so it is administered less frequently There is a significant reduction in the levels of IL-6 and CRP, and it has similar therapeutic results to the two IL-6 inhibitors already on the market (198). In a phase III trial (NCT02760407) in RA, OKZ showed efficacy no less than Adalimumab (199). However, the frequency of TEAEs appears to be dependent on OKZ dose, which may have implications for long-term treatment (198), so more trials are needed to further explore OKZ dose and long-term efficacy once to evaluate the level of RA in treatment.

##### 5.1.2.6 ALX-0061 (12)

ALX-0061 is a 26 kDa bispecific Nanobody targeting IL-6R and HAS, consisting of two sequence-optimized variable domains of VHH antibodies, much smaller than mAbs to facilitate specific binding to IL-6R, so it shows a high binding capacity (Kd of 0.19 ± 0.08 pM for hsIL-6R), whose interaction with HAS effectively prolongs the half-life. ALX-0061 is a novel IL-6 inhibitor that has shown dose-dependent anti-inflammatory effects in IL-6-induced inflammatory models in cynomolgus monkeys (200). In the Phase I/II RA trial, ALX-0061 showed the desired therapeutic outcome, namely well tolerated, no found serious infection and ADA (201), and more trials are needed to confirm its efficacy and safety in the treatment of AIDs.

#### 5.1.3 IL-1

##### 5.1.3.1 Anakinra (13)

Anakinra (ANA; Kineret) is an exogenous recombinant IL-1Ra obtained by mimicking the natural presence of IL-1R antagonist, which is produced by recombinant DNA technology using Eschericlzia coli fermentation. ANA lacks glycosylation and additional amino acid residues in structure, but it does not affect its affinity with IL-1R (binding is comparable to IL-1Ra). ANA restores IL-1/IL-1Ra balance by inhibiting IL-1 activity by competitively binding to IL-1R and has been approved by the FDA for the treatment of RA, early-onset multisystem inflammatory disease (NOMID) in children and adults (202, 203). Compared with other drugs for RA, ANA shows a lower efficacy, in which injection site reactions, high-dose infection, and immunogenicity are the most common adverse reactions, so the overall frequency of use for RA is not very high (202). Clinical trials (NCT01399281, NCT03932344) demonstrated the safety of ANA in the long-term treatment of JIA, with a higher incidence of serious adverse events in the first six months but a decrease thereafter. Existing studies have shown therapeutic promise in refractory brain autoinflammatory-autoimmune diseases, with improved benign responses to symptoms (204). Therefore, the indications for ANA increase, but its adverse effects and safety for long-term use in different diseases have yet to be confirmed.

##### 5.1.3.2 Canakinumab (14)

Canakinumab (CAM; ACZ885; Ilaris^®^), a human IgG1 mAb that specifically targets IL-1β (binding dissociation constant 40 pmol/L) with no cross-reactivity to IL-1α or IL-1Ra, is currently used as an orphan drug for cryopyrin-associated periodic syndromes (CAPSs) and systemic-onset juvenile idiopathic arthritis (sJIA) (>2 years) (205, 206). During treatment with sJIA, CAM rapidly reduced disease activity to delay onset and effectively allowed a gradual decrease in glucocorticoids, and maintained efficacy over a 49-week follow-up period. When reducing the dose or increasing the dosing interval, CAM appears to maintain the clinical remission of CAM in patients with long-term inhibition (206, 207). In terms of safety, patients have mild or moderate adverse reactions in most cases, and the proportion of infections and infestations is the highest, so screening or detection of potential virus should be performed prior to CAM and avoiding combination with TNF inhibitors to reduce the possibility of infection. CAM is not recommended for severely active patients (206). In addition, CAM appears to be effective in the treatment of autoinflammatory diseases (such as familial Mediterranean fever, mevalonate kinase deficiency) (NCT02059291). It has a good prevention control and mitigating effects of flares (based on the PGA score, a comprehensive clinical measure of severity, and the CRP level) and fever to reduce the incidence of disease (208), suggesting that the inhibitory effect of CAM on IL-1 seems to exert a good anti-inflammatory effect, so it is very effective in inflammatory diseases.

##### 5.1.3.3 Rilonacept (15)

Rilonacept (Arcalyst) is a dimeric fusion protein with a molecular weight of 251 kDa composed of the domain of IL-1R and its essential protein and the Fc fraction of human IgG1, which has a high affinity by targeting IL-1β to block IL-1α signaling by acting as a soluble decoy receptor. Rilonacept seems to be used more in the treatment of inflammatory diseases, and has been approved for recurrent pericarditis, CAPSs (209). Studies have shown that Rilonacept has the therapeutic potential of sJIA, but it has not been approved for the treatment of this indication, and it may be that the therapeutic effect is not as good as the previous two IL-1 inhibitors.

##### 5.1.3.4 Gevokizumab (16)

Gevokizumab (XOMA 052) is a humanized anti-IL-1β mAb containing the Fc fraction of IgG1 (high affinity of 300 fM for IL-1β) designed by computer and obtained by ergonomic technology. The inhibitory effect of it on IL-1β-induced IL-6 expression was measured in human lung fibroblasts with an IC_50_ of 4.9 pM, significantly higher than that of Anakinra (210). The advantage of XOMA 052 is that it has a long half-life, allowing it to only need to be administered once a month and improving patient acceptability. XOMA 052 effectively reduces inflammation and improves blood glucose in type 2 diabetes, but has no clear improvement effect on C-peptide levels and β cell function in type 1 diabetes (211, 212). It seems that XOMA 052 is more inclined to the treatment of autoinflammatory diseases, and whether it would have clinical value for AIDs in the future remains to be studied.

#### 5.1.4 IL-17

##### 5.1.4.1 Secukinumab (17)

Secukinumab(SEC; AIN457; Cosentyx) is a fully human IgG1κ mAb that specifically targets IL-17A, which has been approved for the treatment of PsA, AS, psoriasis, moderate to severe plaque psoriasis (PSO), and axSpA (213). SEC reduces immune-mediated inflammatory response by inhibiting the activity of IL-17A and lowers disease activity, which is effective in improving clinical signs and conditions with or without MTX, exhibiting tolerable characteristics consistent with other indications. The most common adverse reactions are nasopharyngitis, headache, diarrhea, and upper respiratory tract infections, with the incidence of severe infections ranging from 1.2/100 to 1.8/100 PY, but it does not appear to increase the risk of viral infection, so the SEC has a long-term safety and therapeutic effect (214, 215). In a phase III comparative trial (NCT02745080) for PsA, SEC did not show a better treatment effect than adalimumab, but had higher treatment retention. Other trials confirmed no significant difference in treatment endpoints (216, 217), which may be that the inhibitory effect on IL-17 and TNF-α did not produce a therapeutic difference in PsA.

##### 5.1.4.2 Ixekizumab (18)

Ixekizumab)IXE; LY2439821; Taltz) is an anti-IL-17A hinge-modified humanized IgG4 mAb with high binding power to human IL-17A or IL-17A/F(Kd<3 pM) to block the binding of IL-17A to IL-17AR. IXE has been approved for PsA, AS, psoriasis, moderate to severe PSO, and axSpA (218). IEX appears to slow disease activity and radiographic disease progression in these diseases, but there is an increased risk of infection. The most common adverse effect is injection site reactions, particularly in the treatment of PsA, which occurs at a significantly higher rate than adalimumab (219); In terms of efficacy, IEX showed superior results over AIN457 and TNF inhibitors in short-term treatment of plaque psoriasis, comparable to brodalumab, risankizumab and guselkumab (220); The efficacy of IEX for AS is similar to that of TNFi and SEC, which could be an alternative for AS patients with an inadequate response to TNFi, but does not appear to be applicable to AS patients with IBD (221).

##### 5.1.4.3 Brodalumab (19)

Brodalumab (AMG827; KHK4827) is a fully human IgG2 mAb that works by blocking the IL-17RA chain of the IL-17 receptor on the cell surface compared to the first two antibodies, and is the first anti-IL-17RA inhibitor to treat psoriasis. The improvement in PsA conditions, safety and good tolerability of AMG827 have been validated in multiple trials (NCT02029495, NCT02024646), which could be used in a larger population (222). However, in the early clinical stages, patient became suicidal after taking AMG827, so it was discontinued and subsequently developed. In the treatment of PSO, AMG827 quickly controls the disease, has a good efficacy, better than ustekinumab, and may be higher than SEC and IXE, which may be because it blocks IL-17 signal more thoroughly. The adverse reactions appear to be consistent with the first two antibodies, such as nasopharyngitis, upper respiratory tract infections, headache and joint pain. However, the FDA issued a black box warning after six patients committed suicide in four clinical trials. So, when using AMG827, it is necessary to pay close attention to the patient’s depressive tendencies and suicide attempts, and this drug cannot be used for patients with such symptoms (223, 224). The existing trial (NCT02985983) also revealed the potential therapeutic effect of AMG827 on axSpA, with long-term safety and efficacy, so its indications may increase subsequently (225).

##### 5.1.4.4 Bimekizumab (20)

Bimekizumab (UCB4940; Bimzelx) is a humanized IgG1/κ mAb produced from CHO cells based on recombinant DNA technology, which selectively inhibits IL-17A and IL-17F to block interaction with IL-17RA/IL-17RC receptor complexes. The binding to IL-17A is higher than that of IL-17F, and the Kd of Human IL-17A, IL-17F and IL-17A/F are 3.2 pM, 23 pM, and 26 pM, respectively. Its inhibitory effect on the pro-inflammatory factor IL-17 allows inflammation to be reversed to improve signs of disease in patients (226, 227). Compared to ustekinumab or adalimumab, Bimekizumab appears to have a faster onset of action and better efficacy in short-term treatment of PSO, better than SEC as well. Its most common adverse reactions are infections, such as upper respiratory tract infections, Candida infections, so preclinical testing, patient screening, and preventive measures are necessary (228, 229). At present, Bimekizumab has been approved to treat moderate to severe PSO, and the treatment of PsA, AS, and axSpA is in clinical trials.

##### 5.1.4.5 Sonelokimab (21)

Sonelokimab (M1095; ALX-0761) is a novel trivalent anti-IL-17A/F bispecific nanobody with a molecular weight of 40 kDa, including three sequence-optimized monovalent camel nanobodies, and specifically targets human IL-17A, IL-17F, and HAS. In a phase I trial of moderate to severe PsA (NCT02156466), M1095 resulted in a significant reduction in inflammatory markers of psoriasis with a favorable safety profile at doses up to 240 mg, and a dose-dependent improvement in the patient’s skin condition was also observed (230). In the Phase II trial for PSO (NCT03384745), M1095 showed significant and rapid clinical efficacy, tolerability and safety similar to SEC. Although its efficacy was confirmed, more clinical data are needed to support subsequent clinical use (231, 232).

##### 5.1.4.6Netakimab (22)

Netakimab(NTK; BCD-085) is a novel recombinant IL-17-resistant humanized IgG1 mAb composed of modified Fc fragments (Fc fragment crystallizable region) and CDR (complementary determinant region), with a strong affinity with IL-17A (Kd 10-12mol/L). Due to its structural characteristics, NTK has the characteristics of low immunogenicity, low toxicity and good tolerance. In phase II trials (NCT03390101, NCT02763111), NTK has good efficacy on PSO and AS with a high clinical response rate, reduces AS activity, improves psoriasis and inflammatory symptoms, and maintains a good safety profile, while a phase III trial of NTK on active AS (NCT03447704) is ongoing (233, 234).

##### 5.1.4.7 Vunakizumab (23)

Vunakizumab (SHR-1314), a humanized IgG1/κ mAb targeting IL-17A, has demonstrated good tolerability and safety in the completed Phase I trials of PSO. In the latest short-term phase II trial (NCT03463187), SHR-1314 has a significantly higher efficacy than placebo, effectively improves the skin of PSO patients with psoriasis area and severity index improvement of at least 75%. Adverse reactions of it are in the known range of (235), providing data support for subsequent clinical trials of larger groups, so SHR-1314 is a potential candidate for the treatment of PSO.

##### 5.1.4.8 CNTO6785 (24)

CNTO6785 is a fully human IgG1λ mAb specifically targeting IL-17A, showing high affinity and selectivity for IL-17A *in vitro*. In a phase II clinical trial for RA with insufficient response to MTX, CNTO6785 has not shown significant clinical efficacy, but it has good tolerability and safety in patients (236), so whether it has a therapeutic effect on immune-mediated diseases needs to be studied later.

##### 5.1.4.9 COVA322 (25)

COVA322 is a bispecific FynomAb obtained by fusing a small anti-IL-17A Fynomer (7 kDa) with adalimumab, which can significantly inhibit TNF and IL-17A (IC_50_ value of 169 pM) *in vivo* and *in vitro* to exert anti-inflammatory effects, and is a potential drug for the treatment of inflammatory diseases, and is currently in the phase 1b/2a trial of PsA (NCT02243787) (237).

##### 5.1.4.10 ABT-122 (26)

ABT-122 (AbbVie) is a novel dual mutant immunoglobulin (DVD-Ig™) molecule targeting both TNF and IL-17A, obtained by combining existing IL-17 antibody with TNF antibody, and the Kd and IC_50_ are in the low pM range. ABT-122 showed good affinity, potency and long half-life in the mouse model of arthritis, and it binding inhibition of the two targets reduced the production of IL-6 by fibroblast-like synovial cells (238). Based on demonstrated acceptable safety in healthy subjects, ABT-122 demonstrated clinical efficacy and safety similar to adalimumab in multiple Phase II trials (NCT02433340, NCT02349451) for patients with RA or PsA, but with little variation, resulting in no further clinical development (239).

##### 5.1.4.11 CJM112 (27)

CJM112 is a novel fully human anti-IL-17A IgG1/κ mAb. Compared with SEC, CJM112 has a higher affinity for human IL-17A, but this does not seem to have a substantial increase in the efficacy of PSO. It shows a clinical effect on the disease, and the efficacy seems to be prolonged with the increase of dose, but it may be accompanied by an increased risk of infection, and it have not shown a therapeutic effect on inflammatory lesions of acne (NCT01828086, NCT0299867) (240, 241), so the clinical use of CJM112 for skin inflammation needs follow-up discussion.

In addition to the above, there are a number of IL-17 antibodies that are still being studied but data have not yet been disclosed, including SCH-90017 in phase I clinical trials and ANB004 (a non-fucosylated ADCC-enhanced anti-human IL17A antibody) has been discovered.

#### 5.1.5 IL-12/IL-23

##### 5.1.5.1 Ustekinumab (28)

Ustekinumab (CNTO 1275; Stelara^®^) is a fully human IgG1/κ mAb obtained through hu-Ig mice technology, which inhibits signaling of the two factors by binding to the IL-12/23 p40 subunit to block its interaction with the cell surface IL-12R β1 receptor. The Kd of single-stranded and heterodimeric human IL-23 is 106 ± 98 pM and 232 ± 23 pM, respectively (242, 243). CNTO 1275 was approved in 2009, and is now used to treat CD, UC, PsA, and PSO, whose safety and efficacy have been evaluated in multiple clinical trials. In a long-term CD clinical trial (NCT01369355), CNTO 1275 kept good safety and tolerability, maintained clinical efficacy for five years, and did not observe an increased risk of adverse effects, which was beneficial for refractory CD with dose escalation. In the treatment of other diseases, CNTO 1275 has also shown long-term efficacy and safety, indicating that it can be used for long-term clinical use (244), but CNTO 1275 is seemly not as effective as Infliximab. CNTO 1275 has also been found to have clinical benefit for SLE.

##### 5.1.5.2 Guselkumab (29)

Guselkumab (CNTO 1959) is a fully human IgG1/λ mAb obtained by MorphoSys HuCAL phage display technology, which binds to the p19 subunit of IL-23 to inhibit the action with the IL-23Rα receptor subunit (Kd 3.3 pM for IL-23 p19) with a higher affinity than CNTO 1275 (242). At present, CNTO 1959 has been used for the treatment of active PsA and moderate to severe PSO. It effectively improves enthesitis, dactylitis, body function, HRQoL in patients with PsA, inhibits imaging progression, and has effective and long-term efficacy, higher than SEC and CNTO 1275, which may be the result of a larger reduction of IL-23/Th17 axis effect factor IL-17 by CNTO 1959 (245). Multiple trials have shown that CNTO 1959 has clinical benefit against CD, having completed Phase II (NCT03466411) (246), and its combination therapy with golimumab (NCT03662542) appears to be more effective in treating UC (247), indicating the potential clinical benefit of CNTO 1959 in the treatment of IBD.

##### 5.1.5.3 Tildrakizumab (30)

Tildrakizumab(MK-3222; SCH 900222) is a humanized IgG1/κ mAb approved in 2018 that specifically targets the p19 subunit of IL-23 and has a binding capacity comparable to CNTO 1275. Tildrakizumab has been used to treat moderate to severe PSO, which has a long-term clinical benefit and is less effective than other IL-12/IL-23 inhibitors, possibly because of its lower affinity with the target but higher than etanercept. In terms of safety, it seems that only MK-3222 has a higher incidence of nasopharyngitis (248). In a real-world study (NCT03718299), Tildrakizumab showed an improvement in HRQoL in PsA patients, and the overall clinical outcome was consistent with other trials (249), so Tildrakizumab could be an alternative option for doctors to treat this disorder.

##### 5.1.5.4 Risankizumab (31)

Risankizumab(BI 655066; ABBV-06) is a high-affinity humanized IgG1/κ mAb with Kd of 21 ± 16 pM and 43 ± 7 pM for single-stranded and heterodimeric human IL-23, respectively. The mechanism and affinity of it appear to be similar to CNTO 1959, and the antibody efficacy *in vitro* and inhibitory effect on skin inflammatory models was also similar, higher than the other two (242). Risankizumab was approved in 2019 for moderate to severe PSO, and its efficacy on PSO is higher than that of CNTO 1275, with better skin clearance than adalimumab. Meanwhile, Risankizumab is in the process of evaluating PsA. A phase III trial (NCT03675308) has shown that CNTO 1275 has significant improvements in joint symptoms, enthesitis, and dactylitis in active PsA, with good efficacy and safety in both single and combination therapies, indicating its therapeutic potential for PsA (250). Other phase III trials (NCT03105102, NCT03105128, NCT03104413) confirmed the clinical efficacy of BI 655066 on CD, which relieved symptoms in patients, with a decrease in inflammatory markers and IL-23 downstream factor IL-22. The overall safety profile of BI 655066 was consistent with previous studies, indicating its potential as a drug for the treatment of CD (251, 252). Therefore, the indications for Risankizumab will increase in the future.

##### 5.1.5.5 Mirikizumab (32)

Mirikizumab (LY3074828; Omvoh^®^), a humanized IgG4 mAb, specifically targets the p19 subunit of IL-23 to inhibit its binding to receptors (21 pmol/L Kd with human IL-23) with an IC_50_ of 82 pmol/L to human IL-23, but it does not affect IL-23 binding to IL-12Rβ1 receptors, which is approved in Japan in 2023 for moderate to severe UC with responding inadequately to conventional therapy (253, 254). In the completed phase III trial of UC, LY3074828 exerted a good clinical response and was significantly more effective than placebo, but randomized discontinuation may lead to disease relapse (254). LY3074828 also completed the evaluation of the phase II trial (NCT02891226) for CD, effectively induced a durable endoscopic response in patients after 12 weeks, and achieved a relatively high endoscopic response rate of (255). LY3074828 has entered the phase III evaluation stage of CD, which is promising as a drug for the treatment of CD. At the same time, there are many phase III trials of UC (NCT03518086, NCT04844606, NCT05509777) in progress (254).

### 5.2 Small molecule inhibitors

#### 5.2.1 TIM1 (37)

SPD304 (**33**) is the first batch of small molecule inhibitors of TNF-α, developed by He et al. SPD304 inhibits the signaling pathway by inducing the subunit decomposition of trimeric TNF-α, but its affinity is general, the physicochemical properties are poor, and not much research has been carried out; JNJ525 (**34**) induces changes in the quaternary structure of TNF to affect the interaction between proteins (256); UCB-9260 (**35**) is a highly bound inhibitor (Kd=13nM) that binds to TNF by structural modification after screening by surface plasmon resonance (SPR) measurement. It stabilizes the asymmetric form of soluble TNF trimer to impair its signal conduction, which appears to improve arthritis in the CAIA model (257). Xiao et al. developed the 1, 5-naphthydine compound - compound 42 (**36**), which has shown biological efficacy in the CAIA model (258). It could be seen that the research of TNF-α small molecule inhibitors is being carried out one after another, and may be developed for clinical use in the future, and TIM1 is a potential small molecule.

TIM1,N-(3-(2-(((3-ethynylphenyl)amino)-1,3-thiazol-4-yl)-2,5-dimethyl 1H-pyrrol-1-yl)-3-methyl-4-oxo-3,4-dihydrophthalazine-1-carboxamide, is a small molecule TNF inhibitor screened based on existing compound libraries and ligand models of SPD304 and JNJ525, which may bind to the central hydrophobic cavity of TNF dimers to block the formation of functional homotrimers (Kd 1.55 ± 0.32 μM with human rhTNF) and low cytotoxicity (LD_50_> 200μM). In the mouse CIA model (a preclinical model of RA), its derivative TIM1c, administered in oral form, showed a similar effect to Etanercept, alleviating RA signs and arthritis, as well as a reduction in inflammatory factors IL-1ß and IL-6, with better anti-inflammatory activity (259). Therefore, the compounds of the TIM1 series have good development prospects.

#### 5.2.2 Madindoline A derivatives (38 39)

IL-6 signaling relies on IL-6 binds to the D2/D3 domains of IL6-Rα and GP130 to form IL-6/IL-6Rα/GP130 heterotrimeric complexes. The natural product Madindoline A (MDL-A) (**38**) is the first selective IL-6 small molecule inhibitor identified, which inhibits the formation of complexes by binding to GP130-D1, but the activity of MDL-A is poor and difficult to obtain, so based on fragments of this structure, Aqel et al. obtained a derivative of MDL-A: MDL-101 (**39**), which inhibited the growth of Th17 cells, the proliferation and function of CD17 T cells and the production of IL-4 *in vitro*, and promoted the development of Treg cells, having the potential to treat multiple sclerosis, but its pharmacokinetic characteristics are poor. Daniel C. et al. used conformational adaptive monosaccharides as an alternative design strategy to obtain a series of carbohydrate-contained compounds, which improved the activity of the compounds and could be used as selective inhibitors of IL-6, requiring further research (260, 261).

#### 5.2.3 LMT-28 (40)

LMT-28, an oxazolidinone derivative, was screened by HepG2 cells transfected with IL-6 stimulation of p-STAT3-Luc, and inhibited the action of IL-6 by targeting gp130 homodimers, with high activity (IC_50_ = 5.9 μM, Kd = 7.4 μM) and low cytotoxicity, and had good pharmacokinetic characteristics (262, 263). In CIA and acute pancreatitis models, LMT-28 exerted a therapeutic effect on arthritis and pancreatitis, decreased arthritis scores, and reduced expression of pro-inflammatory factors to exert anti-inflammatory activity (263). Multiple studies confirmed that LMT-28 reduced gp130 in the IL-6 pathway, phosphorylation of STAT3 with ERK to block signaling, and inhibited of Th17 differentiation, thereby improving arthritis symptoms in CIA, and had a combined effect with metformin (264–266). Therefore, LMT-28 may have potential therapeutic and preventive effects on inflammatory diseases (such as RA, colitis), and may become the first orally available synthetic IL-6 inhibitor.

#### 5.2.4 PF-06650833 (41)

PF-06650833, a highly selective IL-1 receptor-associated kinase 4 (IRAK4) small molecule inhibitor (IC_50_ = 0.2 nM), could be orally absorbed, has good ADME characteristics, and inhibits the production of inflammatory factors (such as TNF, IFN, IL-1, IL-6, IL-12) and macrophage activation in RA, CIA and SLE animal models to reduce inflammatory symptoms (267). Based on good preclinical data, PF-06650833 has completed a phase I trial (NCT02485769, NCT02224651), showing good tolerability and safety (268). It recently has completed a phase II trial (NCT02996500) in RA patients who do not respond adequately to MTX, but the results of which have not yet been disclosed. In addition, two IRAK4 inhibitors, BAY 1834845 (IC_50 =_ 3.4 nM) and BAY1830839 (IC_50_ = 3 nM), have completed multiple clinical trials, the former evaluated in a phase I/II trial (NCT03493269) in psoriasis patients, and the latter completed a multi-dose trial (NCT03540615, NCT03965728).

In addition to the above, there are some small molecule compounds still under development, but the results are not clear. For example, 2, 5-diaminobenzoxazole derivatives show good anti-inflammatory activity in the RA model, in which compound 3e has an inhibition rate of 71.5% on IL-6/STAT3 pathway, and compound 3a has an inhibition rate of IL-1β of 92.1%, and inhibition of these factors improves RA, so such compounds are expected to become drugs for the treatment of RA (269). S011806 is an oral small molecule antagonist of IL-17 developed for the treatment of psoriasis and has entered the phase I clinical stage to explore its safety and pharmacokinetic characteristics, but preclinical data have not yet been disclosed. LEO 153339, as an inhibitor of IL-17, has completed a phase I trial (NCT04883333), but results have not yet been shown.

### 5.3 Natural products

In addition to antibodies and small molecule inhibitors, the therapeutic effect of natural products on AIDs has gradually begun to be revealed. There have been relevant reviews of natural products that have a curative effect on autoimmune arthritis have been summarized. Most of the natural products seem to exert anti-inflammatory activity, inhibit the expression of inflammatory factors to alleviate the disease, and some have entered the evaluation of clinical trials, among which curcumin, resveratrol, triptolian inner fat, green tea is highlighted. These substances have therapeutic effects in a variety of AIDs, with immunomodulatory activity, effectively improve autoimmune inflammation (270–272), and other natural products have been discovered, the following will introduce some new natural products with the potential to treat AIDs.

Aureane-type sesquiterpene tetraketides, isolating from a wetland mud-derived fungus, Myrothecium gramineum (ZLW0801-19), exhibited IL-17A inhibitory activity to regulate immunopathological injury in animal models of experimental autoimmune encephalomyelitis (EAE) and pulmonary hypertension, reducing disease severity, having therapeutic potential for MS and EAE (273).

Prunella vulgaris L. (PV) is a dried fruit spike of the plant Prunella vulgaris L. in the family Lamiaceae, whose inhibition of the HMGB1/TLR9 pathway reduces the proliferation of Th1, Th2, and Th17 cells and the levels of pro-inflammatory factors, thereby improving thyroiditis, and has now been used in China to treat autoimmune thyroiditis (274).

The low-toxicity compound MYMD-1 is a synthetic derivative of tobacco alkaloids, which have been shown to act as an immunomodulator to improve the disease degree and incidence of thyroiditis in autoimmune models, possibly by inhibiting the number of pathogenic Th1 cells and reducing TNF-α production, which seems to have been validated in EAE models while inhibiting the development of EAE. Therefore, MYMD-1 has great potential for the treatment of AIDs (275, 276).

Avocado and soybean unsaponifiables (ASU) are vegetable extracts prepared from fruits and seeds of avocado and soybean oil in a ratio of 1:2. The active ingredients are complex (such as phytosterols, isoflavones), and have a powerful anti-inflammatory effects, so they are effective against scleroderma and IBDs, reduce collagen content and skin fibrosis in scleroderma, and maintain the intestinal barrier in enteritis (277).

Artemisinoids were first discovered as antimalarial drugs, and more experiments have now found that they have a good therapeutic effect on AIDs in preclinical models. Artemisinin derivatives include artemisinin, artesunate, artemether, dihydroartemisinin, and semi-synthetic derivatives (DC32, SM 903, and SM934), which inhibit inflammation in RA to alleviate symptoms, reverse signaling disorders in SLE, and improve clinical signs of IBD, and have therapeutic potential in multiple AIDs (278).

### 5.4 Others

In addition to the above hot targets, there are also some cytokines closely associated with inflammation, including GM-CSF, IL-33, TSLP, and related inhibitors have been gradually developed in AIDs.

Granulocyte-macrophage colony-stimulating factor (GM-CSF), a member of the β common cytokine family, appears to have a pleiotropic modulation of inflammation. During the phase of inflammation resolution, GM-CSF stimulates the proliferation of immunosuppressive bone marrow cells to aid wound healing and tissue repair, while GM-CSF plays a pathogenic role in chronic inflammation. GM-CSF promotes the development of inflammation by acting on innate immune cells (monocytes, neutrophils, macrophages), which are found in RA, IBD, and MS (279, 280). Therefore, a number of GM-CSF inhibitors have been developed for the treatment of AIDs. For example, Tylor et al. demonstrated that IgG1 mAb Namilumab (AMG203), targeting GM-CSF, effectively inhibited macrophage activity in RA patients, exerting beneficial therapeutic effects (281).

IL-33, a member of the IL-1 superfamily, is highly expressed in TH2 cells and mast cells to participate in T cell-mediated immune responses. IL-33 is activated by the orphan receptor ST2 by affecting multiple pathways such as MAPK and NF-κB to increase the release of inflammatory factors, thereby accelerating the pathogenesis of chronic AIDs. Studies have shown that IL-33 and ST2 were abnormally expressed in RA, SLE, SSc, and IBD, and the use of anti-ST2 antibodies reduced the production of IFN-γ, IL-17 and arthritis damage in mice, which indicated the potential role of IL-33 in AIDs (282). Although there is not much research on the use of IL-33 inhibitors, clinical trials conducted by Nnane et al. have demonstrated that IL-33R mAb CNTO 7160 had good PK, PD, and safety in healthy, asthmatic, or atopic dermatitis patients, which supports further clinical studies (283), suggesting that IL-33 inhibitors may be a treatment strategy for subsequent diseases.

Thymic stromal lymphopoietin (TSLP) activates intracellular JAK/STAT, PI3K pathways by binding to receptor TSLPR and mediates the production of inflammatory factors IL-23, IL-17, and IL-4, which may be involved in the pathogenesis of inflammatory AIDs. Although TSLP inhibitors are currently more used in the treatment of allergic diseases, the role of TSLP as a pro-inflammatory mediator in RA has been discovered, and it is also involved in the autoimmune response of some diseases, so the role of TSLP on AIDs would gradually be revealed (284).

## 6 Other inflammatory therapeutic methods

### 6.1 multi-target inhibitors

At present, the development of anti-inflammatory factor inhibitors has focused on single-targeted mAb, but such inhibitors are sometimes not very effective. The pathogenic process of AIDs is often accompanied by the action of multiple inflammatory factors. Studies have shown that simultaneous selection of multiple cytokines for targeted therapy may be faster and better to inhibit disease progression. Fischer et al. (285) confirmed that the combined inhibition of TNF-α and IL-17 would have a synergistic effect on arthritis, which was more effective in reducing the production of cytokines and chemokines. Meanwhile, combined inhibition was more prominent in inhibition of joint inflammation and cartilage damage, maintaining bone homeostasis, which indicates that bispecific antibodies have better efficacy.

Given the benefits of multi-target inhibitors, more research is increasingly biased towards the development of bispecific antibodies (BsAbs). Kang et al. (286) developed a BsAb targeting TNF-α/CXCL10, obtained by combining a single-stranded variable fragment (scFv) resistant to CXCL10 mAb and an Fc region of adalimumab. Compared with adalimumab, BsAb showed similar TNF inhibitory efficacy and anti-arthritis efficacy, but better inhibited the production of inflammatory factors. Whether BsAb is more effective requires further experimental confirmation.

### 6.2 Combined immunotherapy

Nowadays, the therapeutic mechanism of AIDs includes interference with the cell cycle, control of cytokines, inhibition of transport and activation of autoreactive cells and other pathways to inhibit the development of diseases. In most cases, general immunosuppressants are selected for the treatment of AIDs, but due to the lack of antigen specificity, therapeutic effects are not very good, so combination immunotherapy is beginning to be included. The use of antigen-specific immunotherapy (ASIT) in combination with immunomodulators appears to be a good therapeutic strategy, achieved through co-administration or co-delivery (287). Kang et al. demonstrated that the combination of immunosuppressant FK506 and DNA vaccine stimulated regulatory DC, induced antigen-specific Treg, and inhibited the Th17 response, thus preventing EAE (288).

In addition to being used as an alternative to other therapies when other treatments respond inadequately, anti-inflammatory factor inhibitors are commonly used in combination therapies. Numerous studies have shown that compared to biological monotherapy, the combination of it and MTX has a more favorable outcome for AIDs. A phase III clinical trial by Feist et al. (NCT02760433) demonstrated that combination therapy with OKZ and MTX was effective in improving signs and symptoms in RA patients who did not respond adequately to TNFRi (289). While autologous polyclonal Tregs cell therapy has been shown to restore tolerability in T1D patients, the latest experiments have shown that combined it with low-dose IL-2 (ld-IL-2) therapy effectively increased the number of endogenous Tregs, resulting in amplification of Tregs with activation and memory phenotypes, which may be meaningful for adoptive Treg transfer therapy for T1D treatment (290).

### 6.3 Gene therapy

With the gradual deepening of the molecular basis of AIDs, gene therapy has begun to become a potential curative method, achieved by the inactivation or replacement of target genes. At present, there are studies on siRNA treatment of AIDs, indicating that gene therapy has great therapeutic prospects.

siRNA is a novel drug that uses RNA interference to achieve targeted regulation of gene expression, which can effectively silence the gene of interest to treat diseases. Now some siRNAs have been found to be feasible in the treatment of RA. Lee et al. designed a nanocomplex of polymerized siRNA (poly-siRNA), which successfully reduced the production of these factors by targeting the inflammatory genes TNF-α, IL-1, thereby improving arthritis. However, siRNA has many problems such as short half-life, inaccurate positioning, and difficulty in penetrating cell membranes, so it is necessary to achieve the targeting of siRNA with the help of delivery systems. Aldayel et al. developed a TNF-α siRNA nanoparticle formulation that achieved a high encapsulation rate (>90%) to siRNA to increase delivery in inflammatory tissues. In mouse models of arthritis, this formulation demonstrated a potential therapeutic effect of RA with ineffective against MTX (291). Song et al. got a lipid-polymer hybrid nanoparticle FS14-NP/siRNA that packaged siRNA targeting IL-1 to successful delivery to macrophages, showing high gene silencing efficiency. At the same time, its effective accumulation in tissues successfully reduced the expression of inflammatory factors in mice and the bone erosion and cartilage destruction of inflammatory joints (292). These siRNA-nanoparticles show that siRNA interference with inflammatory target genes has an effective therapeutic effect on RA.

In addition, viruses often act as gene delivery vectors. Ebina et al. used adenovirus vectors to transfer APN that exerts anti-inflammatory effects to CIA mice, which subsequently inhibited the development of arthritis. Next, a recombinant AAV vector containing a human TNF-immunoglobulin Fc fusion gene (rAAV2-TNFR: Fc; tgAAC94) has been shown to be effective in inhibiting the development of arthritis in preclinical study, and subsequent clinical trials confirmed good tolerability and safety in patients with RA (293). These all indicate that gene therapy has good potential and may become an important treatment for RA in the future.

## 7 Discussion

The treatment of autoimmune diseases aims to restore homeostasis of the immune system and maintain immune balance by controlling the degree of deviation of the autoimmune response. The treatment strategy has four aspects: changing the immune activation threshold, modulating antigen-specific responses, rebuilding the immune system with autologous or allogeneic stem cells, and preserving the target organ. Antigen-specific therapy aims to induce tolerance to specific antigens. For different AIDs, the choice of drug treatment will be different. Antimalarial drugs, corticosteroids are the preferred treatment strategy for SLE, the first choice for RA treatment is antirheumatoid drugs (such as MTX), and azathioprine is preferred for treatment of AIH. Most of the initial treatment is to choose immunosuppressants, directly targeting B cells and T cell therapy, but these drugs are prone to intolerance, and the patient’s response to it is gradually reduced. Now the drugs for the treatment of AIDs have poor efficacy, too toxic and other problems, so new immunomodulators need to be developed for the treatment of AIDs.

With the increasing research on inflammation of AIDs, it is beginning to realize that inflammation plays an important role in the pathogenesis of AIDs. For example, TNF and IL families play an important pro-inflammatory activity in AIDs, so many treatment strategies for AIDs involve anti-inflammatory therapy, corticosteroids reduce inflammation. The use of cytokine inhibitors (such as TNF, IL-6) and JAK inhibitors for treatment reduces inflammation and pain, and shows better therapeutic efficacy, which is typically used for the treatment of patients who do not respond to basic drugs (39). According to the analysis for biologics in a large number of literature (294, 295), compared with traditional antirheumatic drugs (MTX), glucocorticoids, corticosteroids, biologic disease-modifying antirheumatic drugs (such as TNF-α inhibitors) seem to improve the symptoms and signs of RA patients more quickly, without producing very serious adverse effects, so these drugs have a relative safety profile for RA in the short term. Meanwhile, treatment with biologics and MTX may lead to more effective treatment outcomes. For AS, TNFi has better efficacy and safety than IL and JAK inhibitors, while IL-6 inhibitors have less efficacy and a higher risk of adverse effects, which are not recommended for AS (296). TNFi is also the most effective treatment for CD, among which infliximab leads to better outcomes than adalimumab and certolizumab (297). In the treatment of UC, the small molecule inhibitor Upadacitinib may bring better clinical efficacy than biologics, but it has a high frequency of adverse events. In terms of safety, the anti-integrinin inhibitor Vedolizumab performed best. In biologic-naïve UC, infliximab has the best clinical response rate than adalimumab and golimumab, and it may be the first-line drug of choice for UC (298). Among these anti-inflammatory agents, TNFi is the most widely used, due to both efficacy and safety advantages, but it has not yet had a significant benefit in the treatment of SLE.

However, according to the above analysis of TNFi, most drugs have high immunogenicity, which leads to poor final efficacy. Drugs with lower immunogenicity may have a higher frequency of infection, and the risk of causing other diseases is greatly increased, which may not be conducive to long-term administration. For patients with insufficient response to TNF-α inhibitors, IL inhibitors are selected for treatment. Studies have shown that IL inhibitors have better efficacy in the treatment of POS than TNF-α inhibitors, while some IL-17 inhibitors are more effective in blocking the IL-17 signaling pathway, IL-23 may only be partially inhibited, which may be the reason for the difference in the therapeutic effect of inhibitors (229).

## 8 Prospect

Most of the above anti-inflammatory agents are antibodies, but for the currently developed anti-inflammatory inhibitors, most drugs seem to be injected intravenously, and not all patients achieve a good expected effect. This may be that the pathogenic effect brought by a single action on inflammatory factors is still weak. Meanwhile, some antibodies have the potential for immunogenicity and high production costs. Subsequently, nanobodies begin to enter the market, with small molecular weight, strong specificity, good stability and other characteristics, have great advantages in targeted therapy. However, no such small molecule inhibitors have been used, which is the defect of current anti-inflammatory agent development. Therefore, there are still many unanswered questions in the process of exploring targeting inflammatory pathways to treat AIDs.

In AIDs, the development of inhibitors on inflammatory targets is more focused on biologics, so targeted biological therapies have begun to become a hot treatment strategy. This review describes antibodies (mAbs, BsAbs) in biologics, but in addition to them, antibody-drug conjugates (ADCs) are gradually being developed. For example, Buttgereit et al. (299) demonstrated the ADC ABBV-3373, composed of TNFi adalimumab and a glucocorticoid receptor modulator (GRM), is effective in RA patients (NCT03823391). Compared with single-agent adalimumab, ABBV-3373 improved patients’ symptoms. Therefore, new biologics (BsAb, ADC) are the key research directions of the next generation of biologics, and also provide a new direction for the treatment of AIDs. These targeted inhibitors need to be selected according to the specific disease characteristics of the patient, and the key cytokines for disease pathogenesis are identified, so that specific inhibitors are selected to treat patients more effectively.

For AIDs, other treatment strategies have emerged in addition to drug therapy. Car-T therapy is chimeric antigen receptor T cell immunotherapy, which is currently mainly used as a targeted therapy for tumors, and has good advantages. It has been found that Car-T therapy has therapeutic prospects for AIDs. CARs induce the regulatory role of effector and regulatory T cells in autoimmunity, while CAR-modified T cells effectively kill abnormal immune cells, such as autoantibody-related B cells and plasma cells in AIDs. For example, Jyothi et al. used anti-CD19 CAR-T cells to complete the continuous and effective consumption of B cells in SLE mouse models, and the duration was higher than that of antibodies, and improved the disease of SLE, delayed its occurrence, which played a good preventive role, and showed the potential clinical efficacy of CAR-T therapy on SLE (300). It has subsequently confirmed the feasibility of Car-T therapy in SLE patients, weakening the B-cell-mediated autoimmune response. This relieves the patient’s clinical signs and is highly effective and tolerated (301). This new treatment strategy increases the treatment options for patients with AIDs and may complement immunosuppressants and anti-inflammatory agents. The most common complication of AIDs is interstitial lung disease, so treatment should focus on lung examination, diagnosis, and treatment.

## Author contributions

JS: Writing – review & editing. YX: Writing – original draft. MZ: Writing – original draft. QS: Writing – review & editing. DJ: Writing – original draft.
